# The extracellular polysaccharide determine the physico-chemical surface properties of *Microcystis*

**DOI:** 10.3389/fmicb.2023.1285229

**Published:** 2023-12-05

**Authors:** Haijian Yang, Denghua Wu, Hua Li, Chunxiang Hu

**Affiliations:** ^1^Key Laboratory of Algal Biology, Institute of Hydrobiology, Chinese Academy of Sciences, Wuhan, China; ^2^University of Chinese Academy of Sciences, Beijing, China

**Keywords:** *Microcystis*, cell surface, extracellular polysaccharide, monosaccharide composition, hydrophobicity

## Abstract

*Microcystis* possesses the capacity to form colonies and blooms in lakes and reservoirs worldwide, causing significant ecological challenges in aquatic ecosystems. However, little is known about the determining factors of physico-chemical surface properties that govern the competitive advantage of *Microcystis*. Here, The physico-chemical surface properties of *Microcystis wesenbergii* and *Microcystis aeruginosa*, including specific surface area (SSA), hydrophobicity, zeta potential, and functional groups were investigated. Additionally, the extracellular polysaccharide (EPS) were analyzed. Laboratory-cultured *Microcystis* exhibited hydrophilic, a negative zeta potential and negatively charged. Furthermore, no significant relationship was shown between these properties and the cultivation stage. *Microcystis wesenbergii* exhibited low free energy of cohesion, high surface free energy, high growth rate, and high EPS content during the logarithmic phase. On the other hand, *M. aeruginosa* displayed lower free energy of cohesion, high surface free energy, high EPS content, and high growth rate during the stationary phase. These characteristics contribute to their respective competitive advantage. Furthermore, the relationship between EPS and surface properties was investigated. The polysaccharide component of EPS primarily influenced the SSA and total surface energy of *Microcystis*. Likewise, the protein component of EPS influenced hydrophobicity and surface tension. The polysaccharide composition, including glucuronic acid, xylose, and fructose, mainly influenced surface properties. Additionally, hydrophilic groups such as O–H and P–O–P played a crucial role in determining hydrophobicity in *Microcystis*. This study elucidates that EPS influenced the SSA, hydrophobicity, and surface free energy of *Microcystis* cells, which in turn impact the formation of *Microcystis* blooms and the collection.

## 1 Introduction

Cyanobacterial blooms are a significant environmental issue in aquatic ecosystems (Qu et al., 2014). Common genera that form blooms include *Microcystis*, *Anabaena* (or *Dolichospermum*), *Aphanizomenon*, *Cylindrospermopsis* and others (Huisman et al., 2018). Among these, *Microcystis* is the dominant genus in eutrophic freshwaters worldwide (Xiao et al., 2018). Every year, freshwater lakes with important ecological functions in China, such as Taihu Lake, Chaohu Lake and Dianchi Lake, experience varying degrees of cyanobacterial blooms, with *Microcystis* as the predominant species (Li et al., 2018; Tang et al., 2018). *Microcystis* exhibits a competitive advantage over other organisms in aquatic environments due to its buoyancy regulation, specialized storage strategies, efficient nitrogen uptake, and resistance to zooplankton (Xiao et al., 2018).

In several lakes and reservoirs, the predominant species responsible for the formation of blooms were *M. wesenbergii* and *M. aeruginosa*, among other common *Microcystis* species such as *M. ichthyoblabe* (Zhu et al., 2016)*. Microcystis wesenbergii* and *M. aeruginosa* exhibit alternating blooms in different seasons or depths in the water column, indicating a strong competitive advantage between these two *Microcystis* species (Zhu et al., 2015). In Lake Taihu, *M. wesenbergii* dominated at water temperatures above 27°C, while *M. aeruginosa* dominated at temperature between 22°C and 25°C (Otten and Paerl, 2011). Numerous studies have investigated the relationship among light, temperature, and nutrient competition in *Microcystis* spp. (Yue et al., 2014a,b). For instance, the growth rate of *M. wesenbergii* from Japan remained relatively stable within a temperature range of 20°C–35°C. On the other hand, the growth rate of *M. aeruginosa* showed an increase as the temperature rose. Furthermore, no significant difference in the growth rate between *M. aeruginosa* and *M. wesenbergii* was observed under the same light conditions (Imai et al., 2009). The algal-bacterial relationships of different microcystic algae in nature have also been studied (Zhang et al., 2021; Le et al., 2022). Additionally, the surface characteristics of cells, including EPS, charge, and hydrophobicity play a crucial role in non-specific adhesion of *Microcystis* to various biotic and abiotic surfaces (colony formation) and competitive advantage. However, the surface characteristics of *Microcystis* have received little attention.

Physicochemical properties of microbial cell surfaces such as hydrophobicity, surface tension, contact angle, and surface energy had been studied extensively (Barros et al., 2019). Previous researches have primarily focused on energy microalgae, with the aim of efficient harvesting through flocculation (Zhang et al., 2012). However, limited research has been conducted on cyanobacteria. Liu L. et al. (2016) reported that laboratory-cultured *Microcystis* exists single cells, while wild *Microcystis* forms colonies based on the zeta potential and hydrophobicity. Additionally, the surface features of cells and colony formation are directly influenced by the characteristics of cyanobacterial extracellular polymeric substances (EPS; Liu et al., 2018). The content, composition, and monosaccharide components of EPS directly determine the surface properties of cells, including the presence of non-sugar components such as uronic acids, pyruvic acid, phosphate, and acyl groups. These non-sugar components contribute to the overall negative charge of EPS and provide binding and adsorption properties to these types of polymers (Gupta and Diwan, 2017). Surface adhesions a key factor in determining the self-aggregation capacity of cells (Zhang et al., 2020), and also affects the collection efficiency of cells (Yu et al., 2018). However, the surface characteristics of *Microcystis* are still poorly understood.

This study aimed to examine the cell surface properties of *M. wesenbergii* and *M. aeruginosa*, specifically focusing on cell surface area, hydrophobicity, zeta potential, and surface free energy. Additionally, we investigated the variations in EPS contents and composition at different cultured stages. Our analysis focused on understanding the interactions between cell surface characteristics and EPS. The findings of this study contribute to our understanding of bloom formation (*Microcystis* aggregation) and shed light on the competitive advantage of these two strains. Furthermore, the insights gained from this research may have implications for the development of flocculation collection techniques, such as co-coagulation flotation.

## 2 Materials and methods

### 2.1 *Microcystis* and cultivation

*Microcystis aeruginosa* and *Microcystis wesenbergii* were isolated from water samples collected from Meiliang Bay Meiliang Bay in Lake Taihu in June and November 2008 (*M. aeruginosa* was isolated by Yue Tao, and *M. wesenbergii* was isolated by Li Renhui), which are typical of single populations with different morphotypes. Single colonies were isolated using a light microscopy (Leica, Leica Microsystems, Wetzler, Germany) and purified using a series of BG-11 agar plates until the cultures were axenic. Subsequently, they were identified through 16S rRNA and gvpA-gvpC ITS sequence analysis (Supplementary Figure 5). These colonies were then maintained aseptically in BG11 medium in our laboratory (Yue et al., 2014a,b). The stock cultures were maintained in a sterilized BG11 medium containing 1.5 g NaNO_3_, 40 mg K_2_HPO_4_, 75 mg MgSO_4_ ⋅ 7H_2_O, 20 mg Na_2_CO_3_, 36 mg CaCl_2_ ⋅ 2H_2_O, 6 mg ammonium ferric citrate, 6 mg ammonium citrate monohydrate, 1 mg EDTA, 2.86 μg H_3_BO_3_, 1.81 μg MnCl_2_ ⋅ 4H_2_O, 0.222 μg ZnSO_4_ ⋅ 7H_2_O, 0.39 μg Na_2_MoO_4_ ⋅ 2H_2_O, 0.079 μg CuSO_4_ ⋅ 5H_2_O, 0.050 μg CoCl_2_ ⋅ 6H_2_O in 1 L water. *Microcystis* spp. were cultivated in 5 L Erlenmeyer flasks containing 4 L with continuous illumination under 40 μmol m^−2^ s^−1^ in a temperature-controlled incubator (25°C ± 2°C). The initial inoculum biomass was controlled at about 0.5 mg L^−1^ of Chlorophyll a (*Chl.a*) with a continuous aeration at 0.5 L min^−1^. All the experiments were conducted in triplicate. The microalgal cells were harvested at logarithmic phase after 30-day cultivation and stationary phase after 60-day, and used for the subsequential analyses.

*Microcystis* colonies in the field were isolated and obtained from Lake Taihu in September 2023. The samples were collected using a plankton net positioned approximately 0.5 m above the water surface. Morphological classification using a light microscope was used to identify the *Microcystis* algal strains. Only individual *Microcystis* populations that were free of visible impurities and stray algae were selected. These populations were then rinsed with sterile water and inoculated in test tubes containing BG11 medium at 25°C. After 15 days of incubation, samples were taken for zeta potential and contact angle determination.

### 2.2 Physiology analysis

A 10 mL suspension of microalgal cells was centrifuged at 12,000 rpm for 5 min. The cells sediment was mechanically broken in a mortar with the addition of quartz sand and then resuspended in 2 mL 95% (v/v) ethanol. This suspension was kept in the dark at 4°C for 12 h. Subsequently, the cell suspension was centrifuged for 5 min at 10,000 rpm. The absorbencies of 663, 490, and 384 nm were measured by spectrophotometer, and the content of pigment (mg L^−1^) was calculated using the following equations: *Chl.a* = 1,000 × (1.02 × A663 − 0.027 × A384+ 0.01 × A490)/92.5.

Cell diameters (d) were measured using a microscope (Nikon ECLIPSE 80i, Japan) and dry weight (DW, g L^−1^) was measured by difference method. The Donnan volume (DV, m^3^ g^−1^) was calculated using the following equations: DV = 1/DW × 1,000. The specific surface area (SSA, m^2^ g^−1^) was calculated using the following equations: SSA = S/m = dv × S/V = dv × 4πr^2^/(4/3 × πr^3^) = 6 × DV/d × 10^6^. The surface area per cell (CSA, μm^2^ cell^−1^) were calculated using basic geometric shapes as described by Henderson R. et al. (2008) and dry weight.

### 2.3 Monosaccharide composition and EPS analysis

The EPS matrix was fractionated into the soluble EPS (sEPS, or released polymeric substance, RPS) and bound EPS (bEPS, or capsular polymeric substance, CPS), which are released into the surrounding environment, and which are tightly bound to the cell surface (De Philippis, 1998). The bEPS was also fractionated into the loosely bound EPS (LB-EPS) and tightly bound EPS (TB-EPS). To extract the algae cells and supernatant, 1,000 mL of algal solution was centrifuged at 8000 rpm for 10 min. The algae cells were then washed three times with distilled water and vacuum freeze-dried (Christ ALPHA 1-2 LD plus, Germany) to obtain dry algae. The CPS were extracted from the dry algae (Supplementary material; Supplementary Figures 1–3) with less debris. 100 mg of cells was added into 10 mL of 0.05% NaCl solution, maintained at 60°C for 60 min, and then passed through GF/C Whatman glass microfiber filters (Whatman International Ltd., Maidstone, United Kingdom). Microscopic observation was conducted by staining with India ink to ensure the extract has not been contaminated from intracellular or extracellular substances. Then, the supernatant was dialyzed with distilled water in a Spectrapor dialysis tube with a molecular weight cut-off of 3,500 Daltons (Spectrum China, Shanghai, China). The polysaccharide and protein content in RPS were determined from the supernatant. EPS content refers to the total (mg g^−1^) of RPS and CPS content (Ge et al., 2014). The polysaccharide content was determined using the phenol sulfuric acid method, with glucose as the standard sample, while the protein content was determined using the Bradford assay using bovine serum albumin. The CPS and RPS liquid was isolated to neutral part, acidic I polymers and acidic II polymers (Supplementary material; Supplementary Figure 4).

The monosaccharide composition of EPS was measured using the method by Hokputsa et al. (2003). In briefly, 1 mg of dry weight CPS and RPS samples were subjected to methanolysis using 4 M hydrochloric acid in anhydrous methanol at 80°C for 24 h. To ensure accurate measurements, mannitol was added as an internal standard prior to performing trimethylsilylation. The trimethylsilylated samples were analyzed using gas chromatographic analysis (GC-Orbitrap-MS, Thermo Scientific TRACE 1310-EXACIVE GC). The content of each monosaccharide was calculated based on its content in CPS and RPS.

### 2.4 Surface physicochemical parameters

#### 2.4.1 Zeta potentials analysis

A 10 mL suspension of microalgal cells was prepared by centrifuging at 2,000 g for 20 min. The resulting pellet was washed three times with wate. The pellet was then resuspended in a 0.1 M NaNO_3_ solution and the pH was adjusted from 2 to 8. The zeta potential was determined using a ζ-potential analyzer (Malvern, Zetasizer Nano, ZS90) at room temperature, and the value obtained represents the zeta potential in millivolts (mV).

#### 2.4.2 Hydrophobicity characterization

The hydrophobicity of the cell surface was evaluated using the method described by Rosenberg et al. (1980). The initial content of chlorophyll a (*chl.a* 0) was determined using a 10 mL suspension of microalgal cells. The cells were then resuspended in a phosphate buffer. Subsequently, 2 mL of organic solvents (hexane, ethyl acetate, chloroform) were added to 8 mL of cell suspension. The two-phase system was vortexed for 2 min and allowed to settle for 20 min. The chlorophyll a content of the algal cells in the organic phase was measured as *Chl.a*. The percentage hydrophobicity (H) was calculated as Equation (1):


(1) $$H=\left(Chl.a\;0-Chl.a\right)/Chl.a\;0\times 100\%$$

#### 2.4.3 Contact angles determinations

The cells were suspended in ultrapure water and filtered through a glass fiber membrane with a pore size of 0.45 μm. The membranes were then stored in agar petri dishes containing 1% (w/v) 10% (v/v) glycerol for 2 h to ensure consistent moisture content. Afterward, the membranes were then air-dried for 60 min to achieve a relatively stable contact angle. The contact angle was determined using water (θ_W_), diiodomethane (θ_D_) and formamide (θ_F_) as reference liquids, following the according the method described by Williams et al. (2011).

#### 2.4.4 Free energy of adhesion determinations

The surface free energy of the algae lawns was calculated using the Lifshitz-van Waals/acid–base approach (the LW/AB method). This method decomposes the surface free energy (γ) into two components: Lifshitz-van der Waals component (γ^LW^) and Lewis acid–base component (γ^AB^), which further splits into a Lewis acid component (γ^+^) and a Lewis base component (γ^−^). The LW/AB method can be mathematically expressed as Equations (2)–(4):


(2) $$\gamma_{l}\left(1+\mathrm{cos\theta}\right)=2\sqrt{\left(\gamma_{s}^{LW}\gamma_{l}^{LW}\right)}+2\sqrt{\left(\gamma_{s}^{+}\gamma_{l}^{-}\right)}+2\sqrt{\left(\gamma_{s}^{-}\gamma_{l}^{+}\right)}$$

(3) $$\gamma_{s}^{\mathrm{AB}}=2\sqrt{\left(\gamma_{s}^{+}\gamma_{l}^{-}\right)}$$

(4) $$\gamma =\gamma^{LW}+\gamma^{\mathrm{AB}}$$

where θ is the contact angle, subscripts of “s” and “l” refer to the surface and probe liquid, respectively. In Equation (2), the contact angle of the apolar liquid, diiodomethane, was used to quantify the apolar surface energy component γ_s_^LW^, since γ_l_^−^ and γ_l_^+^ are both equal to zero (van Oss, 1993). Additionally, the contact angles measured with the other two probe liquids, water and formamide, were utilized to calculate the remaining two unknown surface energy parameters, γ_s_^−^ and γ_s_^+^.

According to the Derjaguin, Landau, Verwey, and Overbeek (DLVO) theory, the degree of hydrophilicity and hydrophobicity of the algae surfaces were determined by evaluating their free energy of cohesion (ΔGcoh), as described in Equation (5):


(5) $$\Delta G_{coh}=-2{\left(\sqrt{\gamma_{s}^{LW}}-\sqrt{\gamma_{l}^{LW}}\right)}^{2}+4\left(\sqrt{\gamma_{s}^{+}\gamma_{l}^{-}}+\sqrt{\gamma_{s}^{-}\gamma_{l}^{+}}-\sqrt{\gamma_{s}^{+}\gamma_{s}^{-}}-\sqrt{\gamma_{l}^{+}\gamma_{l}^{-}}\right)$$

where subscripts s and l, respectively, refer to the surface and water. If ΔGcoh < 0, the surface–surface interaction is stronger than the interaction of the surface with water, and the material is treated as hydrophobic. In contrast, the material is hydrophilic if ΔGcoh > 0. Additionally, the free interfacial energy (or total surface tension, G^TOT^) was calculated based on the extended Derjaguin−Landau−Verwey−Overbeek (XDLVO) theory (van Oss, 1993).

#### 2.4.5 Fourier transform infrared spectroscopy

A 10 mL algal solution was centrifuged at 5,000 rpm for 10 min to collect the algal cells. The cells were then vacuum freeze-dried and mixed with KBr powder (1:100, wt/wt). The mixture was processed by pressing and the transmission infrared spectra were recorded using an infrared spectrometer (NEXUS, Thermo Nicolet). The spectrometer had a scanning range of 4,000 to 500 cm^−1^ and a resolution of 2 cm^−1^. The recorded spectra were analyzed using OMNIC software.

### 2.5 Statistical analysis

Results are averages of triplicates, and the values in each graph and table are shown with 5% error bars. t test was performed using SPSS 24.0 package (SPSS, Chicago, IL), with values of 0.05 selected as significance.

## 3 Results

### 3.1 Microalgal growth and cells physico-chemical characterization

#### 3.1.1 Microalgal growth and morphology

**Figure 1:**
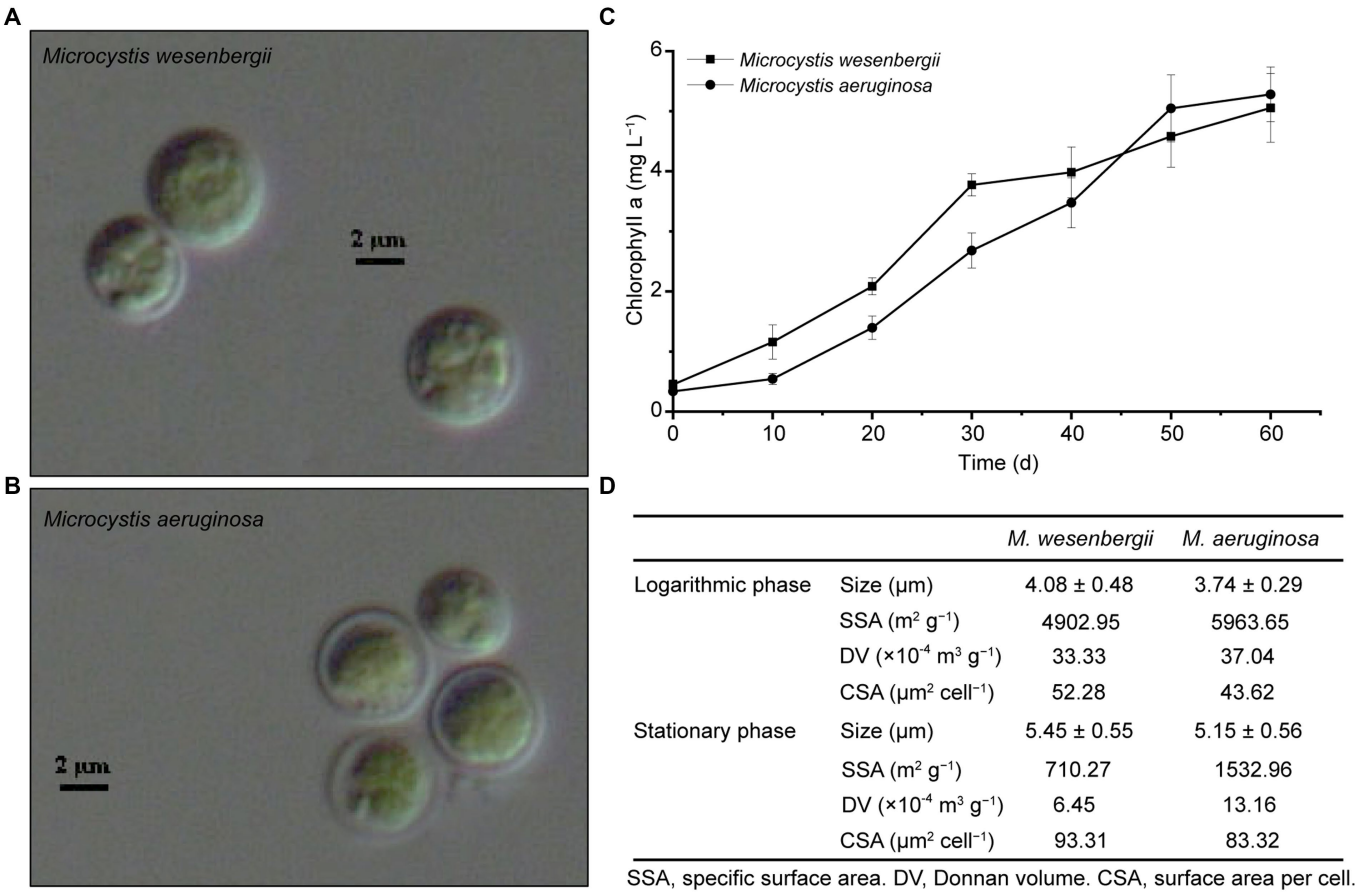
Growth and morphology of *Microcystis*. **(A)** *Microcystis wesenbergii*. **(B)** *Microcystis aeruginosa*. **(C)** Growth curves of *Microcystis*. **(D)** Key cell characterization data for *Microcystis*. L, Logarithmic phase; S, Stationary phases.

*Microcystis* cells are spherical or sub-spherical in shape and are enveloped by a layer of uniform, non-layered, transparent and colorless glue at different stages (Figure 1). As the culture time increased, the size of the cells also tended to increase. Both strains of *Microcystis* exhibited the similar growth rate. *Microcystis wesenbergii* exhibited a higher growth rate during the early stage of logarithmic growth, whereas *M. aeruginosa* exhibited a higher the growth rate during the late logarithmic growth stage. These findings indicated that both strains of *Microcystis* possess relatively strong competitiveness. The SSA and CSV of both strains of *Microcystis* decreased with culture time (Figure 1D). For *M. wesenbergii*, the SSA decreased from 4902.95 to 710.27 m^2^ g^−1^ (a decrease of 85.5%) and for *M. aeruginosa*, the SSA decreased from 5963.65 to 1532.95 m^2^ g^−1^ (a decrease of 74.3%). The CSV decreased by 16.5 and 10.7% for *M. wesenbergii* and *M. aeruginosa*, respectively. These results highlight the significant influence of biomass on SSA.

#### 3.1.2 Cell surface hydrophobicity

**Figure 2:**
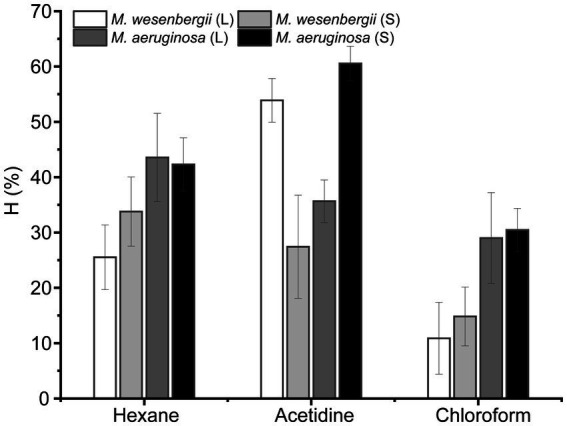
The hydrophobicity of the *Microcystis* cells under different growth phases.

Figure 2 illustrates the hydrophobicity of *Microcystis* in different solvents, namely hexane (a non-polar solvent), ethyl acetate (a polar alkaline solvent), and chloroform (a polar acidic solvent). In polar solvents, the hydrophobicity of *M. wesenbergii* increased with culture time, while in polar alkaline solvents, the hydrophobicity decreased. On the other hand, the hydrophobicity of *M. aeruginosa* showed the opposite trend. During the logarithmic phase, the hydrophobicity of *M. wesenbergii* in non-polar solvents was significantly lower than that of *M. aeruginosa*. However, during the stationary phase, the hydrophobicity of *M. wesenbergii* in non-polar solvents was significantly higher than that of *M. aeruginosa*. Additionally, *Microcystis* had a lower affinity for chloroform (a polar acidic solvent) compared to ethyl acetate (a polar alkaline solvent). The hydrophobicity of *Microcystis* cell surface in hexane was less than 50%, indicating that the cell surface of *Microcystis* was hydrophilic.

#### 3.1.3 Contact angle and surface free energy

Contact angle and surface free energy could directly influence the hydrophobicity. As shown in Table 1, the contact angles with water (θ_W_), formamide (θ_F_), and diiodomethane (θ_D_) on *M. wesenbergii* increased with culture time. For *M. aeruginosa*, θ_w_ increased with culture time, while θ_F_ and θ_D_ decreased. During the logarithmic phase, θ_w_ on *M. wesenbergii* was significantly higher than that of *M. aeruginosa*, whereas θ_F_ and θ_D_ on *M. wesenbergii* were significantly lower than those on *M. aeruginosa*. Conversely, during the stationary phase, θ_w_ on *M. wesenbergii* was not significantly different from that on *M. aeruginosa*, but θ_F_ and θ_D_ were significantly higher. In conclusion, the water contact angles on *Microcystis* were always less than 90°. Additionally, the change of θ_W_ in *Microcystis* from logarithmic phase to stationary phase was more than that of other algal strains (Supplementary Figure 6).

**Table 1:** Contact angles and surface physicochemical properties determined for cyanobacteria.

	Contact angles of the probe liquids (°)			Free energy components (mJ m^−2^)						Zeta (mV)	
Microalgae stain	θ_w_	θ_F_	θ_D_	γ_s_^LW^	γ_s_^AB^	γ_s_	γ_s_^+^	γ_s_^−^	ΔG_coh_	ζ	References
*Microcystis wesenbergii* (L)	26.0 ± 0.5	26.6 ± 0.7	31.2 ± 0.5	43.7 ± 0.2	6.6 ± 0.6	50.3 ± 0.5	0.2 ± 0.0	50.6 ± 0.7	30.3 ± 1.2	−12.07 ± 0.74	
*M. wesenbergii* (S)	32.3 ± 0.6	32.3 ± 0.4	32.1 ± 0.2	43.4 ± 0.1	4.4 ± 0.3	47.7 ± 0.2	0.1 ± 0.0	47.5 ± 0.5	27.6 ± 0.8	−11.93 ± 0.57	
*Microcystis aeruginosa* (L)	22.3 ± 0.3	31.2 ± 0.3	31.9 ± 0.4	43.4 ± 0.2	2.9 ± 0.4	46.3 ± 0.3	0.0 ± 0.0	57.6 ± 0.3	42.0 ± 0.6	−11.32 ± 0.85	
*M. aeruginosa* (S)	32.0 ± 0.3	27.7 ± 0.4	26.8 ± 0.3	45.5 ± 0.1	5.2 ± 0.2	50.7 ± 0.2	0.2 ± 0.0	44.9 ± 0.3	22.1 ± 0.5	−11.85 ± 0.21	This study
*M. aeruginosa* LEGE 91344 (L)	44.8 ± 5.5	37.1 ± 1.6	36.4 ± 8.4	35.9 ± 0.7	10.7 ± 2.8	46.6	0.5 ± 0.4	57.4 ± 7.6	43.4 ± 15.5		Gonçalves et al. (2015)
*S. salina* LEGE 06079 (L)	51.9 ± 8.0	37.3 ± 1.0	73.6 ± 1.1	35.8 ± 0.4	0.0 ± 0.0	35.8	0.0 ± 0.0	22.6 ± 3.2	−10.2 ± 6.6		
*Synechococcus* sp. ATCC 27184 (L)	64	66	61	28.3	0	28.3	0	31.7	10.9	−32.2	Ozkan and Berberoglu (2013)
*A. variabilis* ATCC 29413 (L)	114	71	45	37	0	37	0.3	0	−94.9	−16.80	
*A. variabilis* (S)	97.5 ± 1.5	74 ± 2.1 a	86 ± 3 b	29.19	0	29.19	5.47	0	−55.85	−11.00	Hao et al. (2017)
*Synechocystis* sp.				28.3	3.9	32.2	0.4	10.2	0.1		Volpe and Siboni (1997)
*A. variabilis*				37	0	37	0.2	0	−100.3		

^A^contact angle with glycol; ^b^contact angle with glycerol; L, Logarithmic phase; S, Stationary phases; θ_W_, contact angle with water; θ_F_, contact angle with formamide; θ_D_, contact angle with diiodomethane; γ_s_^LW^, Lifshitz-van der Waals component of the surface free energy; γ_s_^AB^, Lewis acid–base component of the surface free energy; γ_s_, surface free energy; γ_s_^−^, electron donor component; γ_s_^+^, electron acceptor component; ΔG_coh_, the free energy of cohesion; AB, refers to acid–base, i.e., polar component; LW, refers to Lifshitz-van der Waals, i.e., dispersive component; +, refers to electron acceptor parameter; −, refers to electron donor parameter; ξ, Zeta potential (mV).

According to van Oss (1993), microalgae cells were considered hydrophobic when γ_s_^−^ ≥ 28.3 mJ m^−2^ or ΔG_coh_ > 0, and hydrophilic when γ_s_^−^ < 28.3 mJ m^−2^ or ΔG_coh_ < 0. Table 1 shows that the surface of *Microcystis* cells was hydrophilic, which was not directly related to the culture stage. The ΔG_coh_ of *M. wesenbergii* and *M. aeruginosa* decreased with culture time, indicating that cells tended to gather into colonies during the stationary phase. In this phase, the hydrophobicity degree of *M. wesenbergii* (high ΔG_coh_ value) was significantly higher than that of *M. aeruginosa*. However, the change pattern of hydrophilicity reversed in the logarithmic phase. In terms of γ_s_, the γ_s_ values of *M. aeruginosa* increased with culture time, while the γ_s_ values of *M. wesenbergii* decreased. Moreover, the γ_s_ values of both strains of *Microcystis* during the stationary phase were smaller than those during the logarithmic phase. The surface free energy of *M. aeruginosa* was lower than that of *M. wesenbergii* during the logarithmic phase. However, during the stationary phase, the opposite was observed. The nonpolar surface free energy (γ_s_^LW^) of *M. aeruginosa* remained constant throughout the culture time, with no significant difference in γ_s_^LW^ between *M. wesenbergii* and *M. aeruginosa*. These findings suggest that the overall surface free energy and adhesion free energy of *Microcystis* are determined by the polar surface free energy. Additionally, the Supplementary Figure 6 shown that *Microcystis* differed significantly from other algal strains in ΔGcoh and zeta. However, the surface of colonial *Microcystis* cells was hydrophobic (ΔG_coh_ < 0; Supplementary Figure 7).

#### 3.1.4 Physical intercellular interactions

The surface charge of microalgae is an important characteristic of the cell surface. The zeta potential, which refers to the potential of the shear layer on the surface of charged particles, is widely used to describe the electrostatic interaction between colloidal particles and is an important indicator of the stability of colloidal systems. The surface charge of microalgae is influenced by the surface structure and EPS of the cells (Zheng et al., 2019). Microalgal cells produce a significant amount of proteins and sugars both inside and outside the cell membrane, leading to an increase in the negative charges of microalgae. This increase in the absolute value of zeta potential enhances the electrostatic repulsion between microalgae cells, making it difficult for them to coagulate and promoting a more even distribution in the medium. Therefore, the absolute value of the zeta potential can serve as an indicator of the stability of the cell suspension.

**Figure 3:**
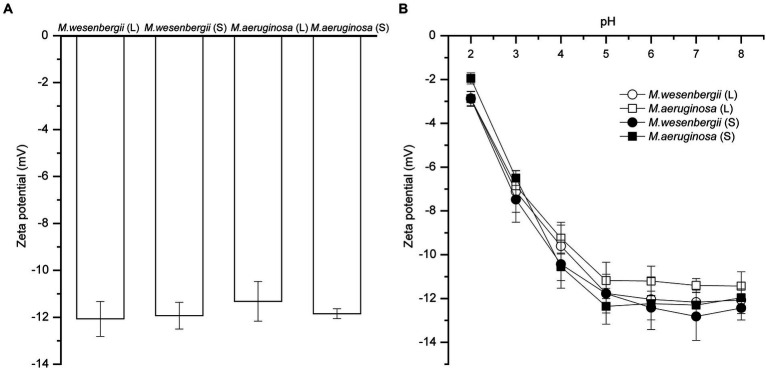
The zeta potential of *Microcystis* under different growth phases **(A)**, and pH **(B)**. L, Logarithmic phase; S, Stationary phases.

Figure 3 shown the zeta potential of *Microcystis* cells at different growth stages and pH levels. The zeta potentials of *Microcystis* were consistently negative throughout different growth stages, indicating the presence of negative charges. Additionally, according to the theory of electric double layer structure (Felix et al., 2014), the binding of OH^−^ ions (and/or release of H^+^ ions) is the main mechanism for charging the particle surface. The negative charges initially increased and then remained stable as the pH levels increased. And, the negative charges of *M. aeruginosa* and *M. wesenbergii* showed no significant change (−11.32~−12.07) from the logarithmic phase to the stationary phase. Thus, the negative charges of *Microcystis* remain relatively stable and do not vary with the duration of growth. Furthermore, there was no significant difference in the zeta potential between these both strains of *Microcystis* (*p* > 0.05). However, the absolute value of zeta was higher in the colonial *Microcystis* compared to the unicellular cells. Additionally, the absolute value of zeta in *M. wesenbergii* was slightly higher than that in *M. aeruginosa*.

### 3.2 Cell surface functional groups under different growth stage

Cell surface functional groups were analyzed using ATR-FTIR spectroscopy (Ferro et al., 2019). There were no marked differences in the spectra characteristic bands of strains corresponding to lipids, including—CH_3_, CH_2_, C–H functional groups [3,050–2,800 and 1,480–1,330 cm^−1^ (Wood et al., 2008)], carbohydrates, including C–O–C, C–OH, and P–O–P functional groups [1,200–950 cm^−1^ (Castro et al., 2010)], nucleic acid backbone conformations, including P=O [1,250–1,000 and 1,000–800 cm^−1^(Liu Y. K. et al., 2016)], and amide I, II, and III, including NH, C–N, C=O [1,660–1,500 cm^−1^, 1,385–1,300 cm^−1^ (Yee et al., 2004)].

**Figure 4:**
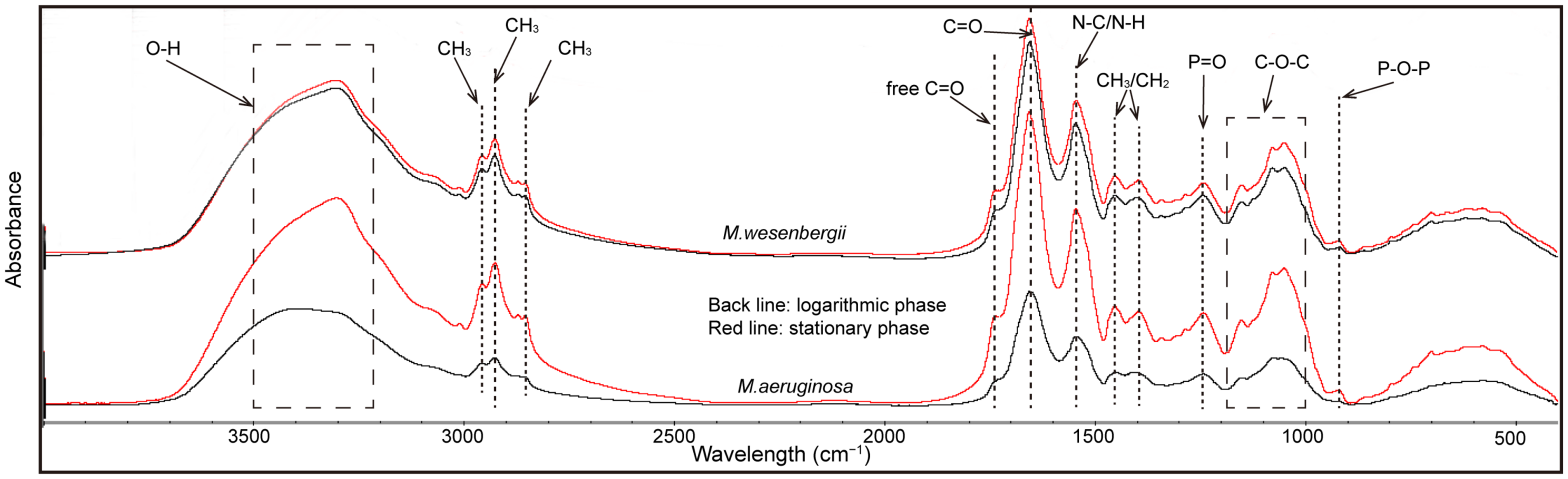
ATR-FTIR spectrum of *Microcystis wesenbergii* and *Microcystis aeruginosa* under different growth phases.

The absorption peaks of various functional groups on the surface of *Microcystis* cells were observed within the spectral ranges of 918–1,737 cm^−1^ and 2,855–3,304 cm^−1^ (Figure 4). Comparatively, the absorption peaks of groups in the stationary phase were stronger than those in the logarithmic phase, indicating the presence of more functional groups involved in cell aggregation during the stationary phase. Notably, the increase in absorption peaks of all groups on *M. aeruginosa* during the stationary phase was significantly higher than that on *M. wesenbergii.* The absorption peak of the O–H group on *M. wesenbergii* remained unchanged throughout the growth stages, whereas the absorption peak of the O–H functional group on *M. aeruginosa* during the stationary phase was significantly higher than that during the logarithmic phase. Additionally, the content of functional groups on *M. aeruginosa* was lower than that on *M. wesenbergii*, with a reversed stability period (Supplementary Table 1). These results showed that more O–H functional groups were present on *M. aeruginosa* cell surface during the stationary phase, which could result in a more negatively charged cell surface. This enhanced negative charge would promote the adsorption of metal ions in the solution, leading to the colony formation. The absorption peak of alkyl group (CH_2_/CH_3_ vibrations) on *M. wesenbergii* remained by the growth stage. However, in the case of *M. aeruginosa*, the alkyl absorption peak during the stationary phase was significantly higher compared to the logarithmic phase. During the logarithmic phase, the alkyl group on *M. aeruginosa* was lower than that on *M. wesenbergii*, whereas the opposite was observed during the stationary phase. The alkyl group plays a crucial role as the primary hydrophobic component. Although the entire cell surface is hydrophilic, the decrease in hydrophilicity during the stationary phase may be attributed to the increased presence of alkyl groups. The absorption peaks of carbonyl groups (C=O) were predominantly observed at 1,622–1,657 and 1,685–1,789 cm^−1^. The range of 1,622–1,657 cm^−1^ could be assigned to the C=O double-bond stretching vibration in the amide I band, while the range of 1,685–1,789 cm^−1^ corresponds to the C=O bond stretching vibration in the free carboxyl group. Peaks representing C–N/N–H group were present at 1,622–1,657 cm^−1^, which were associated with the amide II band. Peaks representing P=O double-bond asymmetric stretching were observed at 1,239 cm^−1^, and are characteristic of nucleic acid or phosphorylated polysaccharides. The absorption peaks corresponding to the C–O–C, C–O–P, and P–O–P ring vibrations of polysaccharides were mainly observed in the range of 900–1,200 cm^−1^, which can be attributed to the mixed peaks of carbohydrates. These changes are similar to the alterations observed in the aforementioned alkyl and OH groups.

### 3.3 EPS and monosaccharide composition

**Figure 5:**
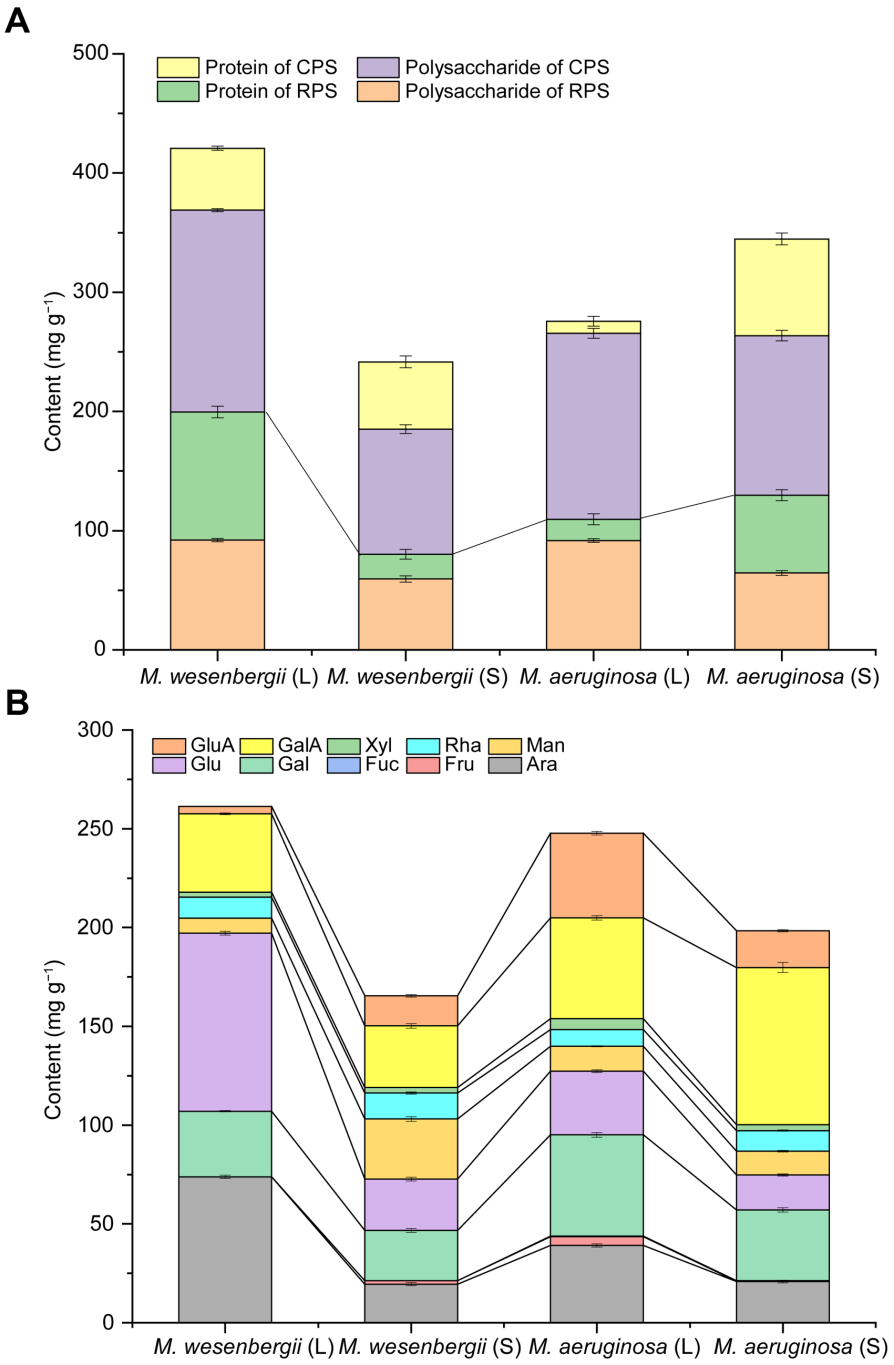
EPS contents **(A)** and monosaccharide composition contents of EPS **(B)** for *Microcystis* at different growth phases. GluA, glucuronic acid; GalA, galacturonic acid; Xyl, xylose; Rha, rhamnose; Man, mannose; Glu, glucose; Gal, galactose; Fuc, Fucose; Fru, fructose; Ara, arabinose. L, Logarithmic phase; S, Stationary phases.

EPS, a viscous substance produced by cyanobacteria cells, serves as a protective barrier against the external environment, such as drought resistance, high salt resistance, ultraviolet radiation resistance, colony formation, and protozoan predation (Pereira et al., 2009; Sun et al., 2020). The dominance of polysaccharide content in EPS was shown in Figure 5A. The EPS content of *M. wesenbergii* decreased significantly with culture time, while the EPS content of *M. aeruginosa* increased significantly. Moreover, during the logarithmic phase, the EPS content of *M. wesenbergii* was significantly higher than that of *M. aeruginosa*, but this trend reversed during the stationary phase. These observations aligned with changes in cell surface free energy and surface functional groups, but contradicts the law of cell adhesion free energy. In term of EPS fraction (Supplementary Figure 4), the neutral fraction of CPS in *Microcystis* cells decreased with culture time, while acidic fractions increased, mainly acidic I polymers. Additionally, the proportion of acidic fraction in EPS was higher during the stationary phase. The main monosaccharide components of the both *Microcystis* strains (Figure 5B; Supplementary Figure 8) include alduronic acid, glucose, arabinose, and galactose. The glucuronic acid abundance of EPS in *M. wesenbergii* increased with culture time, whereas decreased in *M. aeruginosa*. Furthermore, the glucuronic acid abundance of EPS in *M. wesenbergii* was significantly higher than that in *M. aeruginosa* during the logarithmic phase, but becomes equal during the stationary phase. On the other hand, the galacturonic acid concentration increased with culture time in both strains of *Microcystis*. However, the content of galacturonic acid in *M. wesenbergii* consistently remained lower than that in *M. aeruginosa*, regardless of the growth phase.

Figure 5B demonstrated that the total uronic acid content was notably higher during the stationary phase compared to the logarithmic phase. Furthermore, the uronic acid content in *M. wesenbergii* consistently remained lower than that in *M. aeruginosa*. Conversely, the glucose and arabinose contents of EPS during the stable period were significantly lower than those during the logarithmic period. Moreover, the glucose and arabinose contents of EPS in *M. wesenbergii* consistently remained higher than those in *M. aeruginosa*, which contradicted the trend observed for uronic acid. The galactose content of EPS in *M. wesenbergii* increased with culture time, while decreased in *M. aeruginosa*. Moreover, the galactose content in *M. wesenbergii* during the logarithmic phase was significantly higher than that in *M. aeruginosa*. In the stationary phase, the proportion of galactose in both *Microcystis* strains was identical, reflecting the change observed in glucuronic acid. In summary, as the culture time, the monosaccharides that significantly decreased were glucose and arabinose, while galacturonic acid showed a significant increase. Additionally, glucuronic acid and galactose increased in *M. wesenbergii* but decreased in *M. aeruginosa*.

### 3.4 Interrelationship of EPS and functional groups with cell surface properties

**Figure 6:**
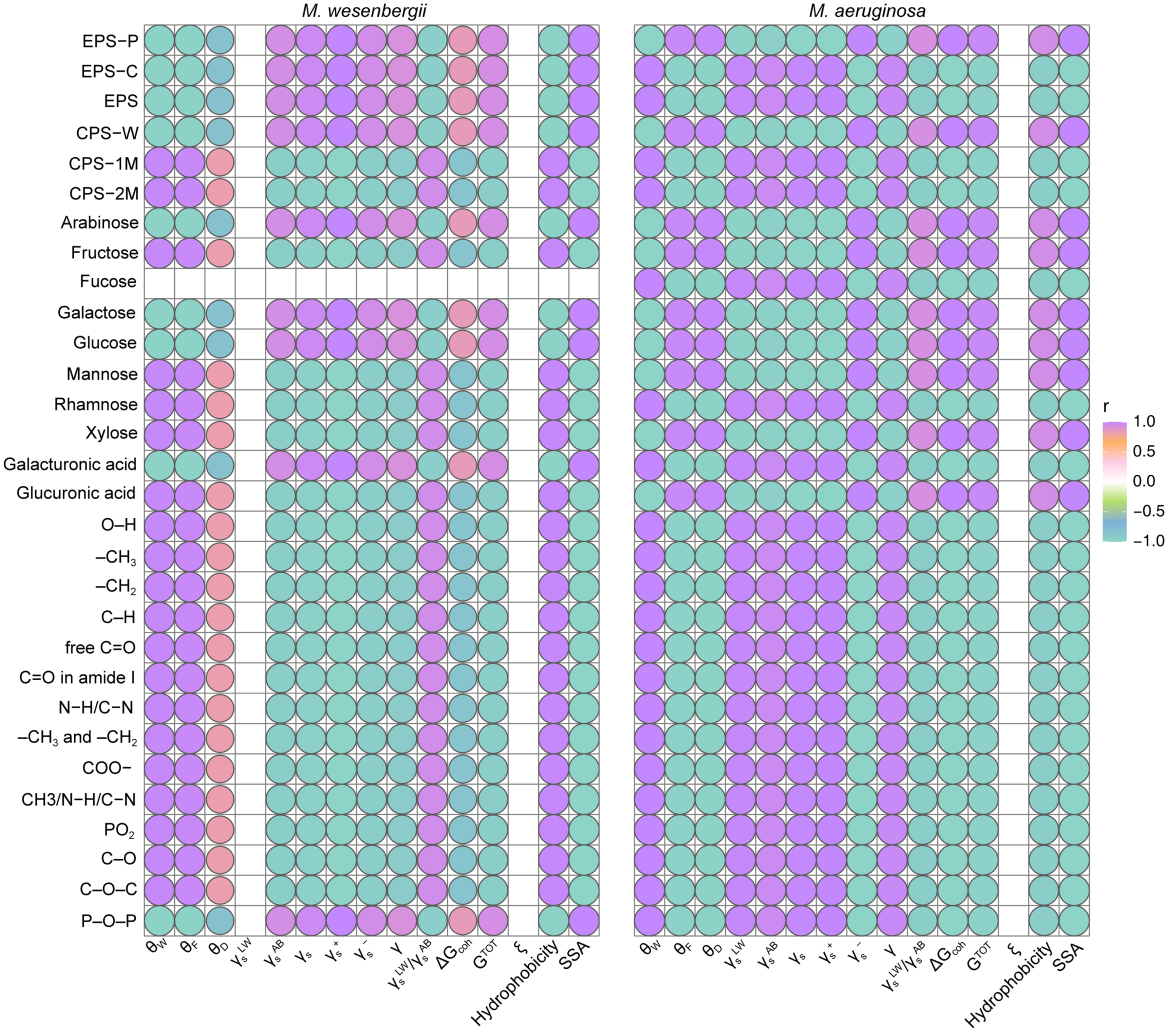
The relation between cell surface characterization and EPS content, composition and functional groups for *Microcystis*. EPS-C, the carbohydrate content in EPS; EPS-P, the protein content in EPS. CPS-W, neutral part in CPS; CPS-1M, acidic I polymers in CPS; CPS-2M, acidic II polymers in CPS. θ_W_, contact angle with water; θ_F_, contact angle with formamide; θ_D_, contact angle with diiodomethane; γ_s_^LW^, Lifshitz-van der Waals component of the surface free energy; γ_s_^AB^, Lewis acid–base component of the surface free energy; γ_s_, surface free energy; γ_s_^−^, electron donor component; γ_s_^+^, electron acceptor component; ΔG_coh_, the free energy of cohesion; AB, refers to acid–base, i.e., polar component; LW, refers to Lifshitz-van der Waals, i.e., dispersive component; +, refers to electron acceptor parameter; −, refers to electron donor parameter; ξ, Zeta potential (mV).

The relationship between the characteristics of EPS (including content, composition, and functional groups) and the cell surface properties of *Microcystis* (such as SSA, charges, surface free energy, surface tension, and hydrophobicity) was depicted in Figure 6. Regarding hydrophobicity, the surface hydrophobicity of *Microcystis* was found to be negatively correlated with EPS content, mainly due to the protein content in EPS. This suggests a higher abundance of hydrophilic amino acids in the protein, along with the arabinose and glucose concentrations in EPS, as well as O–H and P–O–P groups (Supplementary Figure 9). On the other hand, the surface hydrophobicity of *Microcystis* showed a significant positive correlation with xylose, glucuronic acid, and fructose. Notably, *M. wesenbergii* exhibited a significant positive correlation between hydrophobicity and most functional groups, whereas *M. aeruginosa* displayed the opposite trend. Finally, two hydrophilic groups, O–H and P–O–P, were identified as key factors influencing the hydrophobicity of *Microcystis*, with P–O–P primarily found in lipopolysaccharides.

The SSA of *Microcystis* showed a significant positive correlation with the polysaccharide content in EPS and neutral fraction of CPS, as well as with arabinose and galactose concentrations in EPS. Meanwhile, SSA of *Microcystis* exhibited a significant negative correlation with most relevant functional groups and acidic fractions of CPS, as well as with the presence of rhamnose and mannose. The relationship between the surface tension of *Microcystis* and the protein content in EPS, as well as the most relevant functional groups, was found to be significantly positive. On the other hand, there were significant negative correlations with the monosaccharide composition of fructose, xylose, and glucuronic acid. The adhesion free energy of *Microcystis* and G^TOT^ showed a significant positive correlation with the polysaccharide content in EPS, as well as with the monosaccharide composition of fructose, galactose, xylose, and glucuronic acid. On the other hand, there was a significant negative correlation with rhamnose and most functional groups. Therefore, the presence of polysaccharides in EPS primarily influenced the SSA and total surface energy of *Microcystis* cells, while proteins in EPS influenced hydrophobicity and surface tension. Glucuronic acid, xylose, and fructose in the polysaccharide composition mainly impacted the surface properties. The hydrophilic groups O–H and P–O–P were identified as key factors influencing the hydrophobicity of *Microcystis*.

## 4 Discussion

*Microcystis* sp. is a unicellular microorganism with remarkable phenotypic plasticity, which frequently causes the formation of colonies and algal blooms in lakes and reservoirs worldwide (Xiao et al., 2018). In addition to particular adaptation characteristics like buoyancy regulation, cell surface charge and hydrophobicity, notably EPS, also play a role in the development of *Microcystis* colonies or blooms. Additionally, different morphologies of *Microcystis* can result in successional blooms in lakes. This begs the question of how *Microcystis* achieves a competitive edge.

### 4.1 Physico-chemical surface properties of *Microcystis*

In terms of cell growth, size and morphology, *M. wesenbergii* had a higher growth rate in the pre-logarithmic phase and a lower growth rate in the late-logarithmic phase. Additionally, biomass had a significant impact on SSA. The SSA of *Microcystis* was 50 times more than that of the unicellular *Synechococcus* sp. (76 and 88 m^2^ g^−1^; Dittrich and Sibler, 2005). The CSV values of the two *Microcystis* strains were similar to those reported by Henderson R. et al. (2008), indicating that the large SSA can enhance the ability of *Microcystis* to adsorb substances in the liquid phase (Cheng et al., 2019). The SSA was widely recognized as an initial parameter for evaluating the flocculation potential. In the context of *Microcystis* removal through flocculation, a higher SSA value indicates a greater demand for flocculant dosage (Henderson R. et al., 2008). The analysis of contact angle, adhesion free energy, and surface free energy (Table 1) revealed that both strains of *Microcystis* cultivated in the laboratory exhibit hydrophilic characteristics. However, there were noticeable variations in hydrophobicity between *M. wesenbergii* and *M. aeruginosa*, especially in the composition of the non-polar region of the cell surface. The discrepancy may contribute to the differential occurrence of *Microcystis* blooms in different seasons. Notably, during the logarithmic phase, the contact angles of *Microcystis* with solvent were smaller than that of *M. aeruginosa* LEGE 91344 (less than 90°; Gonçalves et al., 2015), and *A. variabilis* (greater than 90°; Volpe and Siboni, 1997; Ozkan and Berberoglu, 2013; Hao et al., 2017). Compared with other freshwater cyanobacteria, *S. salina* LEGE 06079, *Synechocystis* sp., and *A. variabilis* are all hydrophobic (Table 1).

Cell-to-cell interactions can be evaluated using the free energy of adhesion (ΔG_coh_; Yu et al., 2018), which measures the change in free energy before and after adhesion. A higher ΔGcoh suggest a weaker adhesion effect, indicating a stronger repulsive force (or higher mutual absorption) between cells. In this study, *M. wesenbergii* exhibited a lower free energy of adhesion during the logarithmic phase, leading to a higher likelihood of adsorbing substrate and cells. Similarly, *M. aeruginosa* exhibited a lower free energy of adhesion during the stationary phase, making it more prone to adsorb substrate and cells. Both species displayed distinct competitive advantages in this study.

In this study, the zeta potential of *Microcystis* cells remained negative during throughout the cultivation stage and under different pH conditions. This finding is consistent with previous studies on various algal strains, including eukaryotic freshwater single-celled green algae and cyanobacteria (Gonçalves et al., 2015; Xia et al., 2016), marine green algae and diatoms (Ozkan and Berberoglu, 2013), and field *Microcystis* algae (Liu L. et al., 2016). These findings further support the notion that *Microcystis* cells can maintain a stable electronegativity, and that the electrostatic interactions can promote the surface adhesion of cells and metal cations to *Microcystis* cells. However, Liu L. et al. (2016) observed that the absolute value of the zeta potential on the cell surface of *Microcystis* algae was higher during the stationary phase compared to the logarithmic phase, and this difference was influenced by pH. Nonetheless, no significant difference in the absolute values of the zeta potential was observed between the two algal strains during the cultivation period (pH range of from 7 to 8).

### 4.2 EPS and functional groups of *Microcystis*

The cell surface functional groups of *Microcystis* were determined by the composition and content of EPS secreted by the cells. Ionizable functional groups in EPS, such as carboxyl, phosphorus, amino, and hydroxyl groups, can serve as binding sites for divalent cations. These functional groups play a crucial role in determining the properties of the cell surface (Bhunia et al., 2018). The content of hydrophilic groups (including amino groups, hydroxyl groups, carboxyl groups, sulfonic acid groups, aldehyde groups, and phosphoric acid groups) increased during the stationary phase. Similarly, the hydrophobic groups (including hydrocarbon groups, ester groups, and alkanes) also undergo similar changes. These results indicated that the hydrophilicity of the both *Microcystis* strains decreased with culture time. Therefore, the hydrophobic groups play a significant role in the change of hydrophilicity of the algal strains during the culture time. Pringle and Fletcher (1983) demonstrated that an increase in the surface hydrophobicity of microorganisms promoted their mutual approach, facilitating adsorption between microorganisms or with adsorption carriers. In our study, the hydrophobic group contents of *M. aeruginosa* were significantly lower than those of *M. wesenbergii* during the logarithmic phase. However, this trend reversed during the stationary phase, with *M. wesenbergii* exhibiting lower adhesion free energy during the logarithmic phase, making cells easier to adsorb matrix and cells. Conversely, *M. aeruginosa* had lower free adhesion energy during the stationary phase, making cells easier to adsorb matrix and cells. These findings suggested that both strains of *Microcystis* have competitive advantages at different growth stages.

The types of EPS components, such as uronic acid, phosphate, and sulfate (P=O functional group, Figure 4), along with unique connection to sugar groups, give EPS important characteristics (Bhunia et al., 2018). Uronic acids, phosphates, and acetylated sugars primarily contribute to the overall negative charge of EPS, which give EPS its binding and adsorption properties (Gupta and Diwan, 2017). During the stationary phase, the concentration of uronic acid significantly increased, usually in the form of glycosidic bonds. Additionally, the EPS monosaccharide profiles in *Microcystis* were influenced by different culture conditions of distinct algal strains (Forni et al., 1997). However, the polysaccharide content in EPS consistently exceeds that of protein in EPS (Costa et al., 2018). Notably, galacturonic acid was prevalent in *M. flos-aquae*. Among the neutral sugars, fucose, mannose, rhamnose, glucose, uronic acid, and xylose are present in the EPS of *M. flos-aquae*, *M. viridis*, and *M. aeruginosa*, while *M. wesenbergii* contains only uronic acid (Le et al., 2022). The variation in growth rate and the variability in EPS content could potentially shed light on the seasonal succession of *Microcystis*. This may also explain why *M. aeruginosa* blooms were both the larger and last longer in the lake, as well as its predominance in terms of abundance and frequency (Zhai et al., 2013).

### 4.3 Determinants of surface properties of *Microcystis*

The protein content in EPS of unicellular *Microcystis* was hydrophilic in this study. Previous research have shown that an increase in C−(O, N) group content indicated the presence of polysaccharides, which can reduce cell hydrophobicity and increase cell surface energy. Conversely, an increase in C−(C, H) group content indicated the presence of hydrocarbons, which can increase cell hydrophobicity and decrease cell surface energy. Other studies have also demonstrated a relationship between the abundance of phosphodiester-linked and the exposure of hydrophobic regions on the cell surface (Masuoka and Hazen, 1997). In this study, it was found that the C−(O, N) group could effectively reduced the hydrophobicity of *M. aeruginosa*, but had no impact on the hydrophobicity of *M. wesenbergii*. Meanwhile, the C−(C, H) group increased the hydrophobicity of *M. aeruginosa*, but did not have the same effect on *M. wesenbergii*. These findings suggest that the changes in hydrophobicity during different growth stages vary between *M. wesenbergii* and *M. wesenbergii*, and the functional groups involved in these changes also differ. Furthermore, as depicted in Supplementary Figure 5, the O–H and P–O–P groups are shown to play a significant role in influencing the hydrophobicity of *Microcystis*.

The surface free energy of microorganisms is a reflection of the intermolecular force on the surface of microorganisms. It provides insight into the inherent characteristics of microorganisms and serves as a measure of the adhesion between cells and substrates or between cells themselves. Changes in cell surface components and chemical groups can result in alterations in cell free energy. Various substances [such as protein, peptidoglycan, teichoic acid, lipopolysaccharide, EPS, etc. (Liu et al., 2011; Li et al., 2014)] facilitate the interaction of the cell surface with other substances through Lewis acid–base interactions, electrostatic interactions, and hydrophobic interactions. These interactions ultimately influence cell adhesion. Among these components, EPS has been found to have the most significant impact, as supported by numerous studies. For example, EPS can inhibit the adhesion of *Rhodococcus*, while the protein-rich EPS on the surfaces of strains *Sphingobium* and *Micrococcus* has minimal effect on bacterial adhesion to silicone oil (Iwabuchi et al., 2003; Zhang et al., 2011). Sheng and Yu (2006) demonstrated a positive correlation between the γ_b_^AB^ and γb of *Rhodopseudomonas acidophila* with EPS content, as well as a positive correlation with protein/polysaccharide content. In our study, we also observed positive correlations between the γ_s_^AB^ and γ_s_ values and the EPS content on *Microcystis*. Additionally, we found positive correlations between the γ_s_^AB^ and γ_s_ values and the polysaccharide content in EPS on *M. wesenbergii*, as well as a positive correlation with the protein content in EPS on *M. aeruginosa*. Previous studies have already demonstrated the impact of EPS on the formation of *Microcystis* blooms (Zhang et al., 2018; Le et al., 2022).

### 4.4 Determinants of surface properties on colony formation and the contribution of *Microcystis* ecology

Xiao et al. (2017) demonstrated that *Microcystis* colonies formation involves two mechanisms: cell division formation (e.g., *M. ichthyoblabe* colonies formation), and cell adhesion (e.g., *M. wesenbergii* colonies formation). However, cell adhesion leads to faster colony formation. The surface properties of *Microcystis*, such as zeta potential, hydrophobicity, and EPS, play a crucial role in cell adhesion. These properties have a significant impact on the morphology, structure, and function of cyanobacterial colonies (Devasia et al., 1993). Algal cell surface have a negatively charged (zeta < 0), making somewhat hydrophobicity. In the field of *Microcystis* removal, cations are commonly employed as flocculants, due to neutralize the negative surface charge of *Microcystis*. Therefore, Zeta potential as an important index to characterize the surface charge has been applied in wastewater treatment (Henderson R. K. et al., 2008). Furthermore, cations are a factor in the colony formation of *Microcystis*. In addition to neutralize the negative surface charge, divalent Ca^2+^ and Mg^2+^ exhibited a strong binding capability with phenolic −OH, aromatic C − C, and polysaccharide C − O groups to promote forming colonies (Xu et al., 2016; Masumoto et al., 2023). Thus, EPS plays a crucial role in population formation, especially TB-EPS (Xu et al., 2014). Meanwhile, a study indicated that most organic matters were located in the TB–EPS (Xu et al., 2013). This finding aligns with the results of our study, which also observed a higher content of bEPS compared to sEPS, particularly in terms of polysaccharides. Duan et al. (2022) further explain that the protein component of EPS has a negative effect on colony formation, while the sugar component has a positive effect.

The surface properties of colonial *Microcystis* differ from those of unicellular *Microcystis*, and the colonial *Microcystis* cells produce more EPS, which is more conducive to the stabilization of the colonies. Additionally, when *Microcystis* cells form a colony, various environmental factors can influence the physical and chemical properties of the colony. For instance, light and temperature not only impact the photosynthesis of *Microcystis* cells, but also influence parameters such as dissolved oxygen (DO), pH, and Eh (oxidation–reduction potential) of the colony. Then, these factors affect the buoyancy of the colony, where Eh represents the Zeta potential of the colony (Fang et al., 2014). Additionally, influencing factors (nutrient limits and turbulent shear) can induce structural changes within the colonies, enabling to acquire survival strategies (Feng et al., 2020). This study further elucidates that EPS influences the SSA, hydrophobicity, and surface free energy, thereby affecting the formation of *Microcystis* bloom colonies and the efficiency of algal cell collection.

## 5 Conclusion

This study examines the factors that influence the physico-chemical surface properties of *Microcystis*, including EPS polysaccharides, monosaccharide composition and functional groups. The polysaccharide in EPS primarily impacts the surface area and total surface energy of *Microcystis* cells, while the protein in EPS affects hydrophobicity and surface tension. The composition of EPS, specifically glucuronic acid, xylose, and fructose, plays a significant role in determining surface properties. Additionally, during the stationary phase, the content of hydrophilic groups (including amino groups, hydroxyl groups, carboxyl groups, sulfonic acid groups, aldehyde groups, and phosphoric acid groups) increased. Similarly, the hydrophobic groups (including hydrocarbon groups, ester groups, and alkanes) also exhibited similar changes. The hydrophilic group O–H and P–O–P were identified as key groups that influence the hydrophobicity of *Microcystis.* Variations in growth rate, surface properties, and EPS between *M. wesenbergii* and *M. aeruginosa* provide a competitive advantage.

## Data availability statement

The original contributions presented in the study are included in the article/Supplementary material, further inquiries can be directed to the corresponding author.

## Author contributions

HY: Formal analysis, Writing – original draft, Writing – review & editing, Data curation, Software. DW: Data curation, Writing – original draft. HL: Supervision, Writing – review & editing. CH: Conceptualization, Funding acquisition, Writing – review & editing.

## References

[ref1] Barros A. C. Goncalves A. L. Simoes M. (2019). Microalgal/cyanobacterial biofilm formation on selected surfaces: the effects of surface physicochemical properties and culture media composition. J. Appl. Phycol. 31, 375–387. doi: 10.1007/s10811-018-1582-3

[ref2] Bhunia B. Prasad Uday U. S. Oinam G. Mondal A. Bandyopadhyay T. K. Tiwari O. N. (2018). Characterization, genetic regulation and production of cyanobacterial exopolysaccharides and its applicability for heavy metal removal. Carbohydr. Polym. 179, 228–243. doi: 10.1016/j.carbpol.2017.09.091, PMID: 29111047

[ref3] Castro F. D. Sedman J. Ismail A. A. Asadishad B. Tufenkji N. (2010). Effect of dissolved oxygen on two bacterial pathogens examined using ATR-FTIR spectroscopy, microelectrophoresis, and potentiometric titration. Environ. Sci. Technol. 44, 4136–4141. doi: 10.1021/es903692u, PMID: 20438073

[ref4] Cheng Z. Zhang X. Kennes C. Chen J. Chen D. Ye J.. (2019). Differences of cell surface characteristics between the bacterium *pseudomonas veronii* and fungus *Ophiostoma stenoceras* and their different adsorption properties to hydrophobic organic compounds. Sci. Total Environ. 650, 2095–2106. doi: 10.1016/j.scitotenv.2018.09.337 30290351

[ref5] Costa O. Y. A. Raaijmakers J. M. Kuramae E. E. (2018). Microbial extracellular polymeric substances: ecological function and impact on soil aggregation. Front. Microbiol. 9:1636. doi: 10.3389/fmicb.2018.01636, PMID: 30083145 PMC6064872

[ref6] De Philippis R. (1998). Exocellular polysaccharides from cyanobacteria and their possible applications. FEMS Microbiol. Rev. 22, 151–175. doi: 10.1016/s0168-6445(98)00012-6

[ref7] Devasia P. Natarajan K. A. Sathyanarayana D. N. Rao G. R. (1993). Surface chemistry of *Thiobacillus ferrooxidans* relevant to adhesion on mineral surfaces. Appl. Environ. Microbiol. 59, 4051–4055. doi: 10.1128/aem.59.12.4051-4055.1993, PMID: 16349107 PMC195866

[ref8] Dittrich M. Sibler S. (2005). Cell surface groups of two picocyanobacteria strains studied by zeta potential investigations, potentiometric titration, and infrared spectroscopy. J. Colloid Interface Sci. 286, 487–495. doi: 10.1016/j.jcis.2005.01.029, PMID: 15897062

[ref9] Duan Z. Tan X. Zeng Q. (2022). Key physiological traits and chemical properties of extracellular polymeric substances determining colony formation in a cyanobacterium. J. Oceanol. Limnol. 40, 1720–1731. doi: 10.1007/s00343-022-1353-5

[ref10] Fang F. Yang L. Gan L. Guo L. Hu Z. Yuan S.. (2014). DO, pH, and eh microprofiles in cyanobacterial granules from Lake Taihu under different environmental conditions. J. Appl. Phycol. 26, 1689–1699. doi: 10.1007/s10811-013-0211-4

[ref11] Felix C. Yaroshchuk A. Pasupathi S. Pollet B. G. Bondarenko M. P. Kovalchuk V. I.. (2014). Electrophoresis and stability of nano-colloids: history, theory and experimental examples. Adv. Colloid. Interfac. 211, 77–92. doi: 10.1016/j.cis.2014.06.005, PMID: 24997868

[ref12] Feng G. Zhu W. Xue Z. Hu S. Wang R. Zhao S.. (2020). Structural variations increase the upper limit of colony size of *Microcystis*: implications from laboratory cultures and field investigations. J. Phycol. 56, 1676–1686. doi: 10.1111/jpy.13054, PMID: 33448389

[ref13] Ferro L. Gojkovic Z. Gorzsas A. Funk C. (2019). Statistical methods for rapid quantification of proteins, lipids, and carbohydrates in nordic microalgal species using ATR–FTIR spectroscopy. Molecules 24:3237. doi: 10.3390/molecules24183237, PMID: 31492012 PMC6767194

[ref14] Forni C. Telo F. R. Caiola M. G. (1997). Comparative analysis of the polysaccharides produced by different species of *Microcystis* (Chroococcales, Cyanophyta). Phycologia 36, 181–185. doi: 10.2216/i0031-8884-36-3-181.1

[ref15] Ge H. M. Zhang J. Zhou X. P. Xia L. Hu C. X. (2014). Effects of light intensity on components and topographical structures of extracellular polymeric substances from *Microcoleus vaginatus* (Cyanophyceae). Phycologia 53, 167–173. doi: 10.2216/13-163.1

[ref16] Gonçalves A. L. Ferreira C. Loureiro J. A. Pires J. C. M. Simões M. (2015). Surface physicochemical properties of selected single and mixed cultures of microalgae and cyanobacteria and their relationship with sedimentation kinetics. Bioresourc Bioprocess 2:21. doi: 10.1186/s40643-015-0051-y

[ref17] Gupta P. Diwan B. (2017). Bacterial exopolysaccharide mediated heavy metal removal: a review on biosynthesis, mechanism and remediation strategies. Biotechnol. Rep. 13, 58–71. doi: 10.1016/j.btre.2016.12.006, PMID: 28352564 PMC5361134

[ref18] Hao W. Yanpeng L. Zhou S. Xiangying R. Wenjun Z. Jun L. (2017). Surface characteristics of microalgae and their effects on harvesting performance by air flotation. Int. J. Agr. Biol. Eng. 10, 125–133. doi: 10.3965/j.ijabe.20171001.2698

[ref19] Henderson R. Parsons S. A. Jefferson B. (2008). The impact of algal properties and pre-oxidation on solid-liquid separation of algae. Water Res. 42, 1827–1845. doi: 10.1016/j.watres.2007.11.039, PMID: 18261761

[ref20] Henderson R. K. Parsons S. A. Jefferson B. (2008). Successful removal of algae through the control of zeta potential. Sep. Sci. Technol. 43, 1653–1666. doi: 10.1080/01496390801973771

[ref21] Hokputsa S. Hu C. X. Paulsen B. S. Harding S. E. (2003). A physico-chemical comparative study on extracellular carbohydrate polymers from five desert algae. Carbohydr. Polym. 54, 27–32. doi: 10.1016/S0144-8617(03)00136-X

[ref22] Huisman J. Codd G. A. Paerl H. W. Ibelings B. W. Verspagen J. M. H. Visser P. M. (2018). Cyanobacterial blooms. Nat. Rev. Microbiol. 16, 471–483. doi: 10.1038/s41579-018-0040-1 29946124

[ref23] Imai H. Chang K.-H. Nakano S.-I. (2009). “Growth responses of harmful algal species *Microcystis* (Cyanophyceae) under various environmental conditions” in Interdisciplinary studies on environmental chemistry—Environmental research in Asia. eds. Obayashi Y. Isobe T. Subramanian A. Suzuki S. Tanabe S. (Tokyo, Japan: TerraPub), 269–275.

[ref24] Iwabuchi N. Sunairi M. Anzai H. Morisaki H. Nakajima M. (2003). Relationships among colony morphotypes, cell-surface properties and bacterial adhesion to substrata in *Rhodococcus*. Colloid. Surface. B. 30, 51–60. doi: 10.1016/S0927-7765(03)00036-5

[ref26] Le V. V. Srivastava A. Ko S. R. Ahn C. Y. Oh H. M. (2022). *Microcystis* colony formation: extracellular polymeric substance, associated microorganisms, and its application. Bioresour. Technol. 360:127610. doi: 10.1016/j.biortech.2022.127610 35840029

[ref27] Li L. Wang Z. Rietveld L. C. Gao N. Hu J. Yin D.. (2014). Comparison of the effects of extracellular and intracellular organic matter extracted from *Microcystis aeruginosa* on ultrafiltration membrane fouling: dynamics and mechanisms. Environ. Sci. Technol. 48, 14549–14557. doi: 10.1021/es5035365, PMID: 25402823

[ref28] Li M. Xiao M. Zhang P. Hamilton D. P. (2018). Morphospecies-dependent disaggregation of colonies of the cyanobacterium *Microcystis* under high turbulent mixing. Water Res. 141, 340–348. doi: 10.1016/j.watres.2018.05.017, PMID: 29804020

[ref29] Liu Y. X. Alessi D. S. Owttrim G. W. Kenney J. P. L. Zhou Q. X. Lalonde S. V.. (2016). Cell surface acid-base properties of the cyanobacterium *Synechococcus*: influences of nitrogen source, growth phase and N:P ratios. Geochim. Cosmochim. Acta 187, 179–194. doi: 10.1016/j.gca.2016.05.023

[ref30] Liu L. Z. Huang Q. Qin B. Q. (2018). Characteristics and roles of *Microcystis* extracellular polymeric substances (EPS) in cyanobacterial blooms: a short review. J. Freshwater Ecol. 33, 183–193. doi: 10.1080/02705060.2017.1391722

[ref31] Liu L. Huang Q. Qin B. Zhu G. Wu P. Wu Y. (2016). Characterizing cell surface of blooming *Microcystis* in Lake Taihu, China. Water Sci. Technol. 73, 2731–2738. doi: 10.2166/wst.2016.069, PMID: 27232410

[ref32] Liu J. Lu L. J. Huang X. F. Shang J. J. Li M. X. Xu J. C.. (2011). Relationship between surface physicochemical properties and its demulsifying ability of an alkaliphilic strain of *Alcaligenes* sp. S-XJ-1. Process Biochem. 46, 1456–1461. doi: 10.1016/j.procbio.2011.03.018

[ref33] Masumoto A. Amano Y. Machida M. (2023). Enhancement of cyanobacterial blooms buoyancy by controlling extracellular polysaccharides content and cation concentration under light-limited condition. Int. J. Environ. Sci. Technol. doi: 10.1007/s13762-023-05014-4

[ref34] Masuoka J. Hazen K. C. (1997). Cell wall protein mannosylation determines *Candida albicans* cell surface hydrophobicity. Microbiology 143, 3015–3021. doi: 10.1099/00221287-143-9-3015, PMID: 9308183

[ref35] Otten T. G. Paerl H. W. (2011). Phylogenetic inference of colony isolates comprising seasonal *Microcystis* blooms in Lake Taihu, China. Microb. Ecol. 62, 907–918. doi: 10.1007/s00248-011-9884-x, PMID: 21667196

[ref36] Ozkan A. Berberoglu H. (2013). Physico-chemical surface properties of microalgae. Colloid. Surface. B. 112, 287–293. doi: 10.1016/j.colsurfb.2013.08.001, PMID: 24001448

[ref37] Pereira S. Zille A. Micheletti E. Moradas-Ferreira P. De Philippis R. Tamagnini P. (2009). Complexity of cyanobacterial exopolysaccharides: composition, structures, inducing factors and putative genes involved in their biosynthesis and assembly. FEMS Microbiol. Rev. 33, 917–941. doi: 10.1111/j.1574-6976.2009.00183.x, PMID: 19453747

[ref38] Pringle J. H. Fletcher M. (1983). Influence of substratum wettability on attachment of freshwater bacteria to solid surfaces. Appl. Environ. Microbiol. 45, 811–817. doi: 10.1128/aem.45.3.811-817.1983, PMID: 16346243 PMC242376

[ref39] Qu M. Lefebvre D. D. Wang Y. Qu Y. Zhu D. Ren W. (2014). Algal blooms: proactive strategy. Science 346, 175–176. doi: 10.1126/science.346.6206.175-b, PMID: 25301608

[ref40] Rosenberg M. Gutnick D. Rosenberg E. (1980). Adherence of bacteria to hydrocarbons: a simple method for measuring cell-surface hydrophobicity. FEMS Microbiol. Lett. 9, 29–33. doi: 10.1016/0378-1097(80)90106-8

[ref41] Sheng G. P. Yu H. Q. (2006). Relationship between the extracellular polymeric substances and surface characteristics of *Rhodopseudomonas acidophila*. Appl. Microbiol. Biotechnol. 72, 126–131. doi: 10.1007/s00253-005-0225-1, PMID: 16292527

[ref42] Sun F. Zhang H. Qian A. Yu H. Xu C. Pan R.. (2020). The influence of extracellular polymeric substances on the coagulation process of cyanobacteria. Sci. Total Environ. 720:137573. doi: 10.1016/j.scitotenv.2020.137573, PMID: 32143047

[ref43] Tang X. Krausfeldt L. E. Shao K. LeCleir G. R. Stough J. M. A. Gao G.. (2018). Seasonal gene expression and the Ecophysiological implications of toxic *Microcystis aeruginosa* blooms in Lake Taihu. Environ. Sci. Technol. 52, 11049–11059. doi: 10.1021/acs.est.8b01066, PMID: 30168717

[ref44] van Oss C. J. (1993). Acid—base interfacial interactions in aqueous media. Colloids Surf. A Physicochem. Eng. Asp. 78, 1–49. doi: 10.1016/0927-7757(93)80308-2

[ref45] Volpe C. D. Siboni S. (1997). Some reflections on Acid–Base solid surface free energy theories. J. Colloid Interface Sci. 195, 121–136. doi: 10.1006/jcis.1997.5124 9441613

[ref46] Williams D. Kuhn A. O'Bryon T. Konarik M. Huskey J. (2011). Contact angle measurements using cellphone cameras to implement the Bikerman method. Galvanotechnik 102, 1718–1725.

[ref47] Wood B. R. Chernenko T. Matthäus C. Diem M. Chong C. Bernhard U.. (2008). Shedding new light on the molecular architecture of oocytes using a combination of synchrotron Fourier transform-infrared and Raman spectroscopic mapping. Anal. Chem. 80, 9065–9072. doi: 10.1021/ac8015483, PMID: 18983174 PMC2761072

[ref48] Xia L. Li H. Q. Song S. X. (2016). Cell surface characterization of some oleaginous green algae. J. Appl. Phycol. 28, 2323–2332. doi: 10.1007/s10811-015-0768-1

[ref49] Xiao M. Li M. Reynolds C. S. (2018). Colony formation in the cyanobacterium *Microcystis*. Biol. Rev. Camb. Philos. Soc. 93, 1399–1420. doi: 10.1111/brv.12401, PMID: 29473286

[ref50] Xiao M. Willis A. Burford M. A. Li M. (2017). Review: a meta-analysis comparing cell-division and cell-adhesion in *Microcystis* colony formation. Harmful Algae 67, 85–91. doi: 10.1016/j.hal.2017.06.007, PMID: 28755723

[ref51] Xu H. Jiang H. Yu G. Yang L. (2014). Towards understanding the role of extracellular polymeric substances in cyanobacterial *Microcystis* aggregation and mucilaginous bloom formation. Chemosphere 117, 815–822. doi: 10.1016/j.chemosphere.2014.10.061, PMID: 25465953

[ref52] Xu H. Lv H. Liu X. Wang P. Jiang H. (2016). Electrolyte cations binding with extracellular polymeric substances enhanced *Microcystis* aggregation: implication for *Microcystis* bloom formation in eutrophic freshwater lakes. Environ. Sci. Technol. 50, 9034–9043. doi: 10.1021/acs.est.6b00129, PMID: 27502019

[ref53] Xu H. Yu G. Jiang H. (2013). Investigation on extracellular polymeric substances from mucilaginous cyanobacterial blooms in eutrophic freshwater lakes. Chemosphere 93, 75–81. doi: 10.1016/j.chemosphere.2013.04.077 23726883

[ref54] Yee N. Benning L. G. Phoenix V. R. Ferris F. G. (2004). Characterization of metal-cyanobacteria sorption reactions: a combined macroscopic and infrared spectroscopic investigation. Environ. Sci. Technol. 38, 775–782. doi: 10.1021/es0346680 14968864

[ref55] Yu G. Cai X. Shen L. Chen J. Hong H. Lin H.. (2018). A novel integrated method for quantification of interfacial interactions between two rough bioparticles. J. Colloid Interface Sci. 516, 295–303. doi: 10.1016/j.jcis.2018.01.075, PMID: 29408116

[ref56] Yue T. Zhang D. Hu C. X. (2014a). Utilization of phosphorus in four forms of the three dominant *Microcystis* morphospecies in Lake Taihu. J. Lake Sci. 26, 379–384. doi: 10.18307/2014.0307

[ref57] Yue T. Zhang D. L. Hu C. X. (2014b). Comparative studies on phosphate utilization of two bloom-forming *Microcystis* spp. (cyanobacteria) isolated from Lake Taihu (China). J. Appl. Phycol. 26, 333–339. doi: 10.1007/s10811-013-0067-7

[ref58] Zhai C. M. Song S. Zou S. H. Liu C. H. Xue Y. R. (2013). The mechanism of competition between two bloom-forming *Microcystis* species. Freshw. Biol. 58, 1831–1839. doi: 10.1111/fwb.12172

[ref59] Zhang X. Amendola P. Hewson J. C. Sommerfeld M. Hu Q. (2012). Influence of growth phase on harvesting of Chlorella zofingiensis by dissolved air flotation. Bioresour. Technol. 116, 477–484. doi: 10.1016/j.biortech.2012.04.002, PMID: 22541950

[ref60] Zhang P. L. Chen M. Z. Zhang Y. P. Li Y. M. Lu S. Li P. F. (2018). Autoaggregation and adhesion abilities in bacteria associated with colonies of *Microcystis*. Hydrobiologia 823, 205–216. doi: 10.1007/s10750-018-3706-9

[ref61] Zhang J. Y. Nawaz M. Z. Zhu D. C. Yan W. Alghamdi H. A. Lu Z. H. (2021). Diversity, seasonal succession and host specificity of bacteria associated with cyanobacterial aggregates in a freshwater lake. Environ. Technol. Innov. 24:101988. doi: 10.1016/j.eti.2021.101988

[ref62] Zhang Y. Wang F. Yang X. Gu C. Kengara F. O. Hong Q.. (2011). Extracellular polymeric substances enhanced mass transfer of polycyclic aromatic hydrocarbons in the two-liquid-phase system for biodegradation. Appl. Microbiol. Biotechnol. 90, 1063–1071. doi: 10.1007/s00253-011-3134-5 21327962

[ref63] Zhang X. Yuan H. Wang Y. Guan L. Zeng Z. Jiang Z.. (2020). Cell surface energy affects the structure of microalgal biofilm. Langmuir 36, 3057–3063. doi: 10.1021/acs.langmuir.0c00274, PMID: 32160744

[ref64] Zheng Y. Huang Y. Xia A. Qian F. Wei C. (2019). A rapid inoculation method for microalgae biofilm cultivation based on microalgae-microalgae co-flocculation and zeta-potential adjustment. Bioresour. Technol. 278, 272–278. doi: 10.1016/j.biortech.2019.01.083, PMID: 30708330

[ref65] Zhu W. Li M. Dai X. X. Xiao M. (2015). Differences in vertical distribution of *Microcystis* morphospecies composition in a shallow hypertrophic Lake (lake Taihu, China). Environ. Earth Sci. 73, 5721–5730. doi: 10.1007/s12665-014-3826-0

[ref25] Zhu W. Zhou X. Chen H. Gao L. Xiao M. Li M. (2016). High nutrient concentration and temperature alleviated formation of large colonies of *Microcystis*: Evidence from field investigations and laboratory experiments. Water Res. 101, 167–175. doi: 10.1016/j.watres.2016.05.080 27262121

