# Supplementary material for: The extracellular polysaccharide determine the physico-chemical surface properties of Microcystis
Source: Front Microbiol. 2023 Dec 5;14:1285229. doi: 10.3389/fmicb.2023.1285229 (PMC10732508; doi:10.3389/fmicb.2023.1285229)
Supplement: Supplementary file 1 [file Data_Sheet_1.docx]

Supplementary Material

# Supplementary Material and Methods

## CPS extraction for different duration

The CPS were extracted from the wet algae. 10 ml of cells was added into 10 ml of 0.05% NaCl solution, maintained at 60 °C for 15, 30, 45, 60, 75, 90 min, and then passed through GF/C Whatman glass microfiber filters (Whatman International Ltd, Maidstone UK). The supernatant was dialyzed with distilled water in a Spectrapor dialysis tube with a molecular weight cut-off of 3500 Daltons (Spectrum China, Shanghai, China).

## Isolation and purification of EPS

The CPS and RPS liquid was concentrated to 50 mL under vacuum condition using a BÜCHI Rotavapor R-210 (BÜCHI Labortechnik AG, Flawil, Switzerland) at 36 ℃. The concentrated solution was then spiked directly onto a DEAE-Sepharose fast flow gel column (50 × 5 cm, Bomei), following the method described by Hu et al. (2003). The column was first eluted with distilled water at 2 mL min^−1^, for collection of the neutral part, then eluted with 1.0 M NaCl to collect the acidic I polymers, followed by 2.0 M NaCl to get the acidic II polymers. All part were dialyzed successively against running tap water and distilled water in a Spectrapor dialysis tube with a molecular weight cut-off of 3500, and finally freeze dried. The relative proportions of the components are calculated by dry weight.

# Supplementary Result

Treatment period affected the CPS yield (Supplementary Figure 1). Polysaccharides content increased with duration. Polysaccharides are the major components in the CPS of *Microcystis*; therefore, the increased polysaccharides content indicates an enhanced CPS extraction. Extending the treatment period also increased the EPS yield. Polysaccharides levels peaked when *Microcystis* cultures were treated at 60 °C for 60 min.


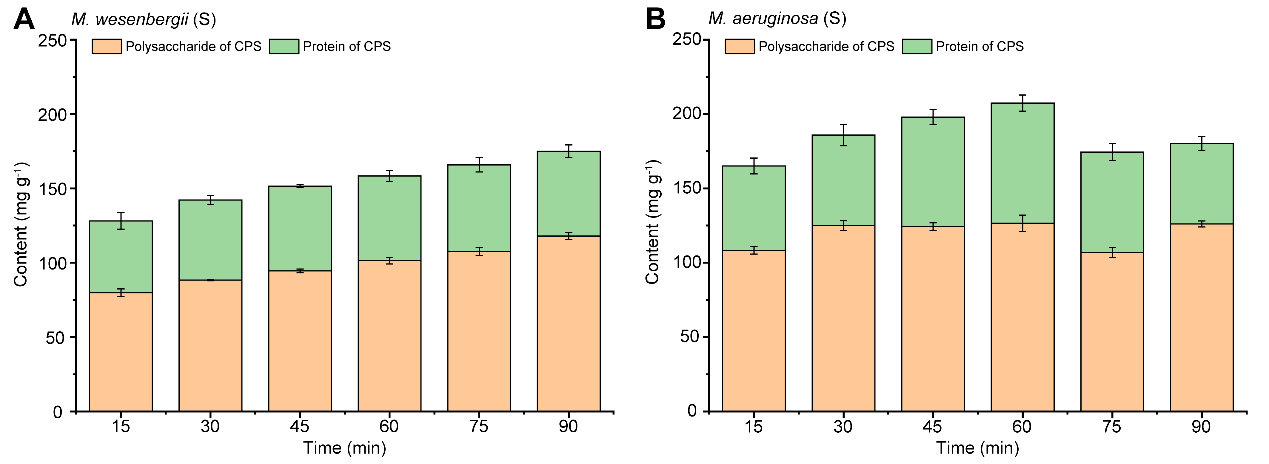


**Supplementary Figure 1.** Polysaccharide and protein contents in CPS of *M. wesenbergii* and *M. aeruginosa* (B) with wet cells derived from different duration.

While using wet cells or dry cells methods facilitated CPS extraction from *Microcystis*, The CPS yield and composition were not significantly different from wet cells and dry cells (Supplementary Figure 2). Throughout the vacuum freeze-dried cells and CPS extraction process, the cells undergo only one relysis, to ensure the integrity of the cells. Additionally, this method compresses excess space to extract more cells, thereby providing a sufficient amount of EPS for elution.


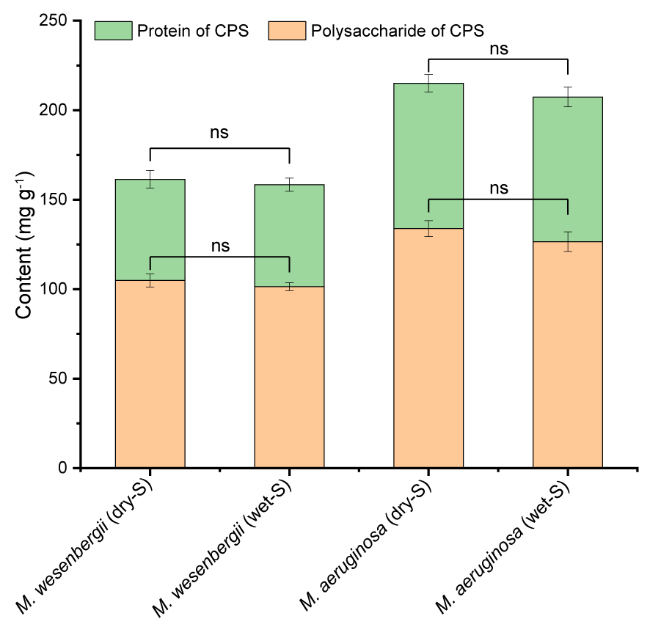


**Supplementary Figure 2.** Comparison of wet and dry cells for the extraction of CPS from *Microcystis* in terms of CPS yield (polysaccharide and protein) and the ratio of polysaccharide to protein that indicates the relative content of polysaccharides in the extract. *Microcystis* (dry-S or wet-S), CPS was extracted using the dry cells or wet cells of *Microcystis* cells during the stationary phase. ns indicates no significant difference (*Microcystis* (dry-s) vs. *Microcystis* (wet-S)).

Cell lysis commonly produces cell debris, and induces the release of cellular inclusion. To determine whether CPS extraction from dry cells induces more cell lysis and whether CPS is extracted completely, we observed cell fragmentation under a microscope (Supplementary Figure 3). Less debris was also found in dry cells.


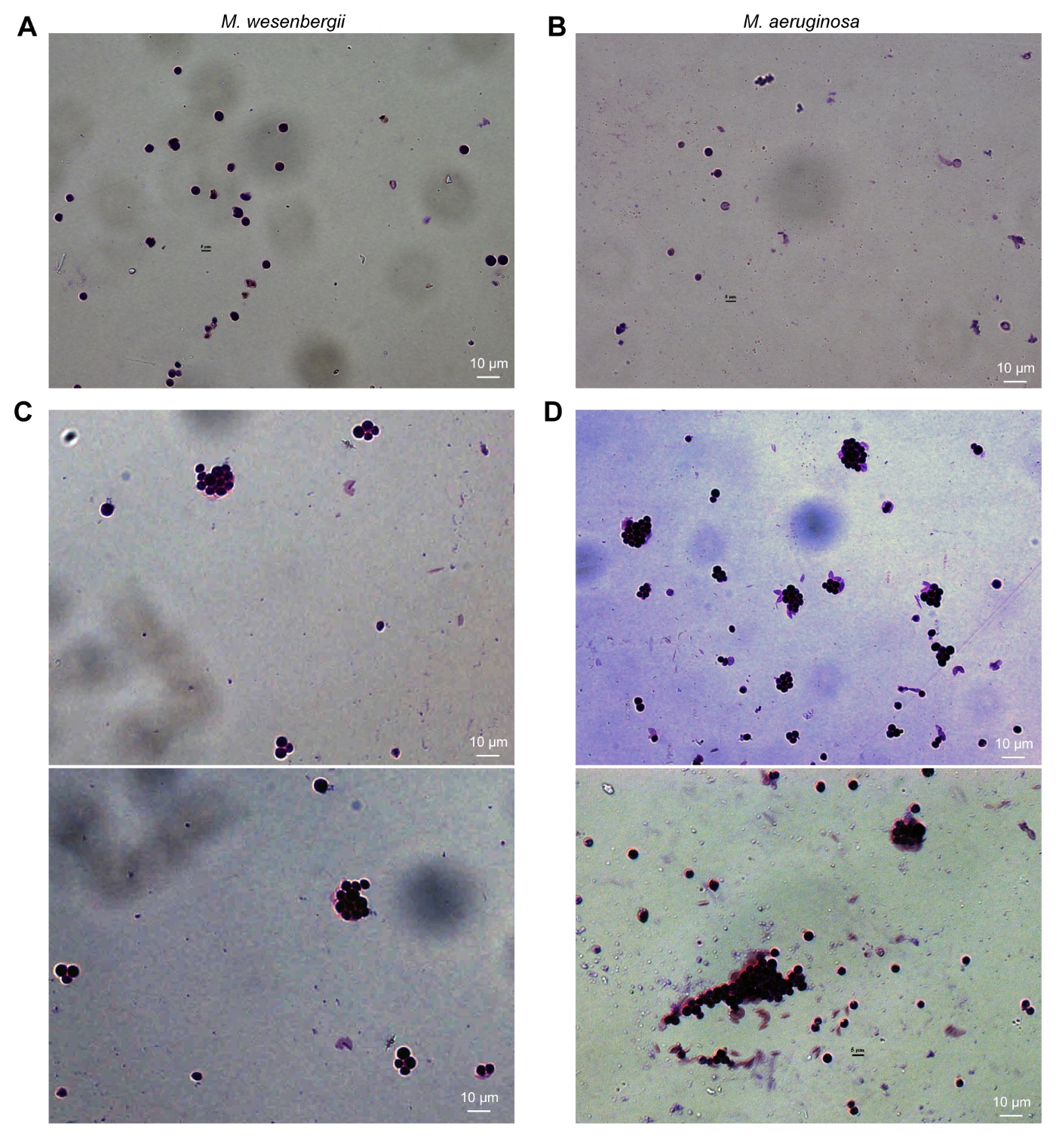


**Supplementary Figure 3.** Changes in the morphological characteristics of *Microcystis* cells after the extraction of CPS under different methods. Wet cells treatment (A and B), Dry cells treatment (C and D).

The EPSs produced by all organisms were separated into one neutral and two acidic fractions by anion exchange chromatography and the amounts for each fraction (Supplementary Figure 4). The neutral fraction of CPS in *Microcystis* cells decreased with culture time, while acidic fractions increased, mainly acidic I polymers. Additionally, the proportion of acidic fraction in EPS was higher during the stationary phase.


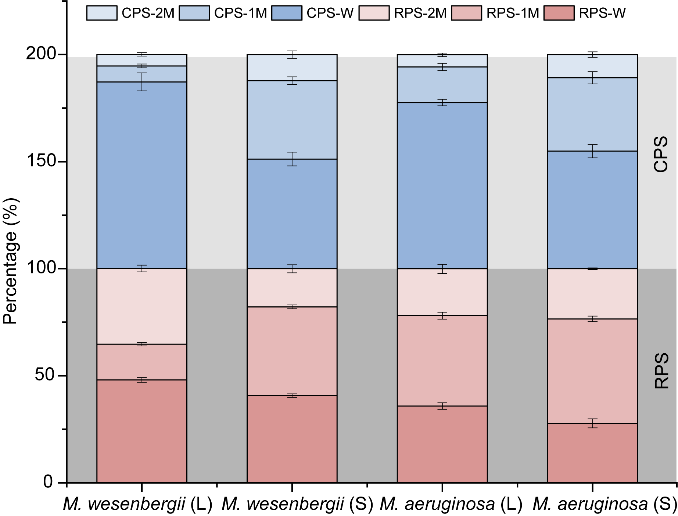


**Supplementary Figure 4.** CPS and RPS fractions of of *Microcystis*. -W, water eluted, neutral part. -1M, 1 M NaCl eluted, acidic I polymers. -2M, 2 M NaCl eluted, acidic II polymers.

# Supplementary Figures and Tables

## Supplementary Tables

**Supplementary Table 1** The assignments of principal vibrational bands (Yee et al., 2004; Meade et al., 2007; Castro et al., 2010).

| Wavelength (cm^−1^) | Functional group assignment |
| --- | --- |
| 3304-3432 | Antisymmetric stretching of O–H vibrations in water |
| 2957-2986 | Vs Stretching of –CH_3_ functional groups |
| 2926-2939 | Stretching of -CH_2_ functional groups |
| 2855 | Stretching of C–H functional groups |
| 1685-1789 | C=O of protonated carboxylic acid groups |
| 1622-1657 | Stretching of C=O in amide I, associated with proteins |
| 1516-1564 | N–H bending and C–N stretching in amide II, associated with proteins |
| 1438-1478 | Bending of CH_3_ and das CH_2_ of lipids or proteins |
| 1397-1432 | stretching vibration of COO^－^ from carboxyl groups |
| 1312-1385 | bending stretching of CH_3_, vibration of C–N, and bending of N–H of proteins (amide III) and fatty acids |
| 1232-1273 | asymmetric stretching vibration of P=O of the phosphodiester backbone of nucleic acid (DNA and RNA), free phosphate, or monoester phosphate functional groups, symmetric stretching of C–O of COO– groups |
| 1134-1152 | Vibration of C–OH, C–O from carbohydrates |
| 1108-1128 | Vibration of C–OH, C–O from phosphodiester and carbohydrates |
| 1028-1098 | Mixed vibrational modes of carbohydrates; PO_2_^-^,C–O–P, C–OH, C–O–C, and P–O–P of polysaccharides |
| 996-997 | C–O, C–C from carbohydrates, nucleic acids, or amino acids |
| 895-928 | P–O–P ring vibrations of polysaccharides |

## Supplementary Figures


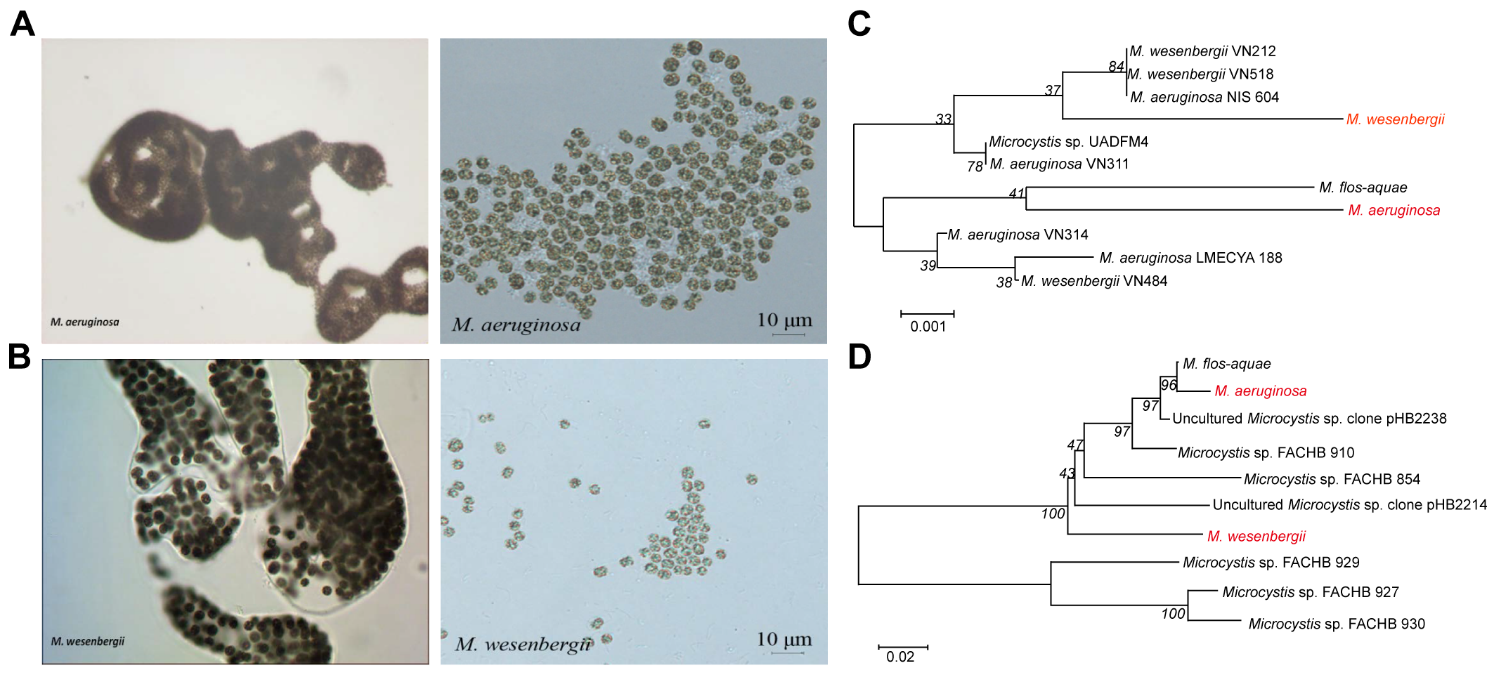


**Supplementary Figure 5.** Microscope images and of the *M. aeruginosa* (A) and *M. wesenbergii* (B) morphospecie, and phylogeny for 16S rRNA (C) and *gvpA-gvpC* (D) intergenic gene sequences.


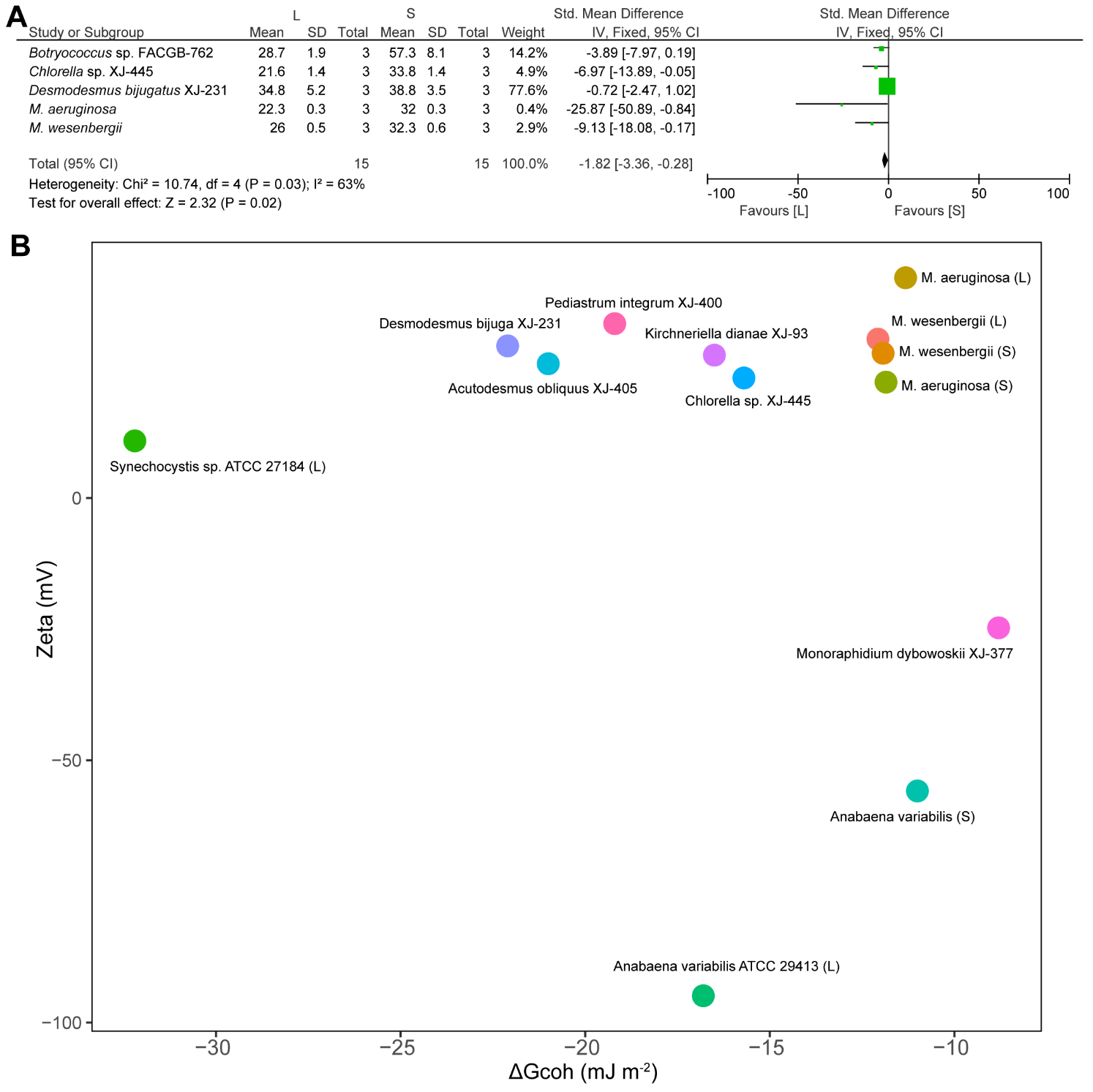


**Supplementary Figure 6.** Comparison of surface properties of *Microcystis* with other algal strains (Table 1 and Xia et al., 2016). A, the mate anlysis of microalgae between the logarithmic growth and stationary phas; B, the zeta potential and free energy of cohesion (ΔGcoh) of microalgae.


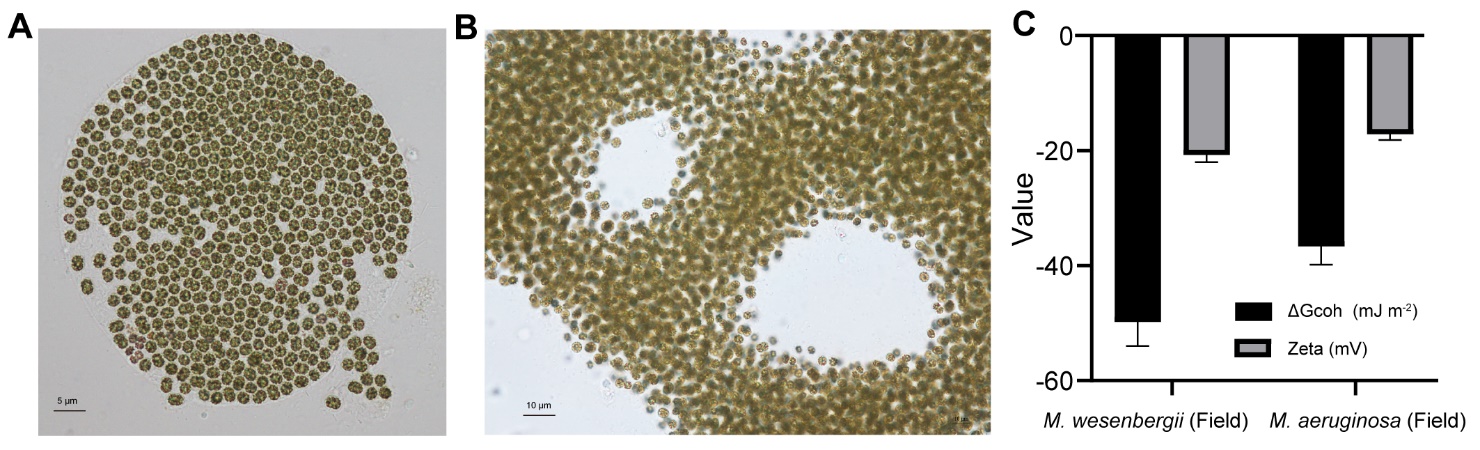


**Supplementary Figure 7.** Microscopic (A, *M. wesenbergii;*B*, M. aeruginosa*) and surface properties of *Microcystis* in the field.

**Supplementary Figure 8.** Monosaccharide composition (%) of EPS for *Microcystis* at different growth phases. GluA, glucuronic acid; GalA, galacturonic acid; Xyl, xylose; Rha, rhamnose; Man, mannose; Glu, glucose; Gal, galactose; Fuc，Fucose;Fru, fructose; Ara,arabinose; L, Logarithmic phase; S, Stationary phases.


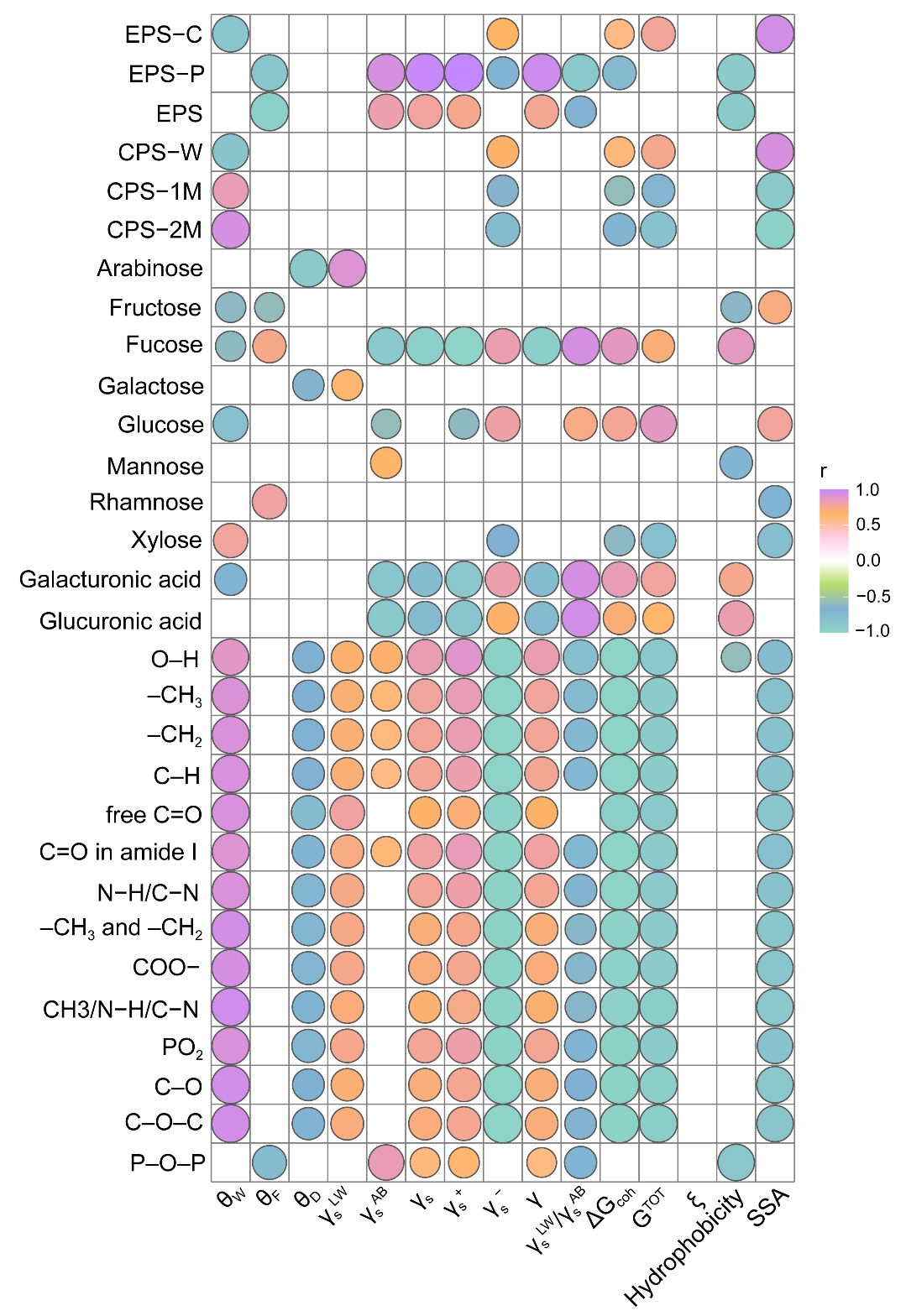


**Supplementary Figure 9.** The relationship between cell surface characterization and EPS content, composition and functional groups for *Microcystis*. EPS-C, the carbohydrate content in EPS; EPS-P, the protein content in EPS. CPS-W, neutral part in CPS; CPS -1M, acidic I polymers in CPS; CPS -2M, acidic II polymers in CPS. θ_W_, contact angle with water; θ_F_, contact angle with formamide; θ_D_, contact angle with diiodomethane; γ_s_^LW^, Lifshitz-van der Waals component of the surface free energy; γ_s_^AB^, Lewis acid-base component of the surface free energy; γ_s_, surface free energy; γ_s_^−^, electron donor component; γ_s_^+^, electron acceptor component; ΔG_coh_, the free energy of cohesion; AB, refers toacid–base,i.e.polarcomponent; LW, refers toLifshitz–vanderWaals,i.e.dispersivecomponent; +, refers to electron acceptor parameter; −, refers to electron donor parameter; ξ, Zeta potential (mV).

**Supplementary References**

Hu, C., Liu, Y., Paulsen, B.S., Petersen, D., and Klaveness, D. (2003). Extracellular carbohydrate polymers from five desert soil algae with different cohesion in the stabilization of fine sand grain. *Carbohyd. Polym.* 54, 33-42. doi: 10.1016/S0144-8617(03)00135-8

Castro, F.D., Sedman, J., Ismail, A.A., Asadishad, B., and Tufenkji, N. (2010). Effect of dissolved oxygen on two bacterial pathogens examined using ATR-FTIR spectroscopy, microelectrophoresis, and potentiometric titration. *Environ. Sci. Technol.* 44(11)**,** 4136-4141. doi: 10.1021/es903692u.

Meade, A.D., Lyng, F.M., Knief, P., and Byrne, H.J. (2007). Growth substrate induced functional changes elucidated by FTIR and Raman spectroscopy in in–vitro cultured human keratinocytes. *Anal. Bioanal. Chem.* 387(5)**,** 1717-1728. doi: 10.1007/s00216-006-0876-5.

Xia, L., Li, H.Q., and Song, S.X. (2016). Cell surface characterization of some oleaginous green algae. *J. Appl. Phycol.* 28, 2323-2332. doi: 10.1007/s10811-015-0768-1.

Yee, N., Benning, L.G., Phoenix, V.R., and Ferris, F.G. (2004). Characterization of metal-cyanobacteria sorption reactions: a combined macroscopic and infrared spectroscopic investigation. *Environ. Sci. Technol.* 38(3)**,** 775-782. doi: 10.1021/es0346680.
